# The FANCD2-FANCI heterodimer coordinates chromatin openness and cell cycle progression throughout DNA double-strand break repair

**DOI:** 10.1016/j.celrep.2025.116830

**Published:** 2026-01-07

**Authors:** Christine M. Joyce, Julien Bacal, Soham P. Chowdhury, Andrew N. Brown, Amy K. Wang, Carmen Cruz, Kameron Bains, Zachary N. Rodriguez, Nathan J. McCormick, Yaara Tzadikario, Katherine U. Tavasoli, Brooke M. Gardner, Chris D. Richardson

**Affiliations:** 1 Department of Molecular, Cellular, and Developmental Biology, University of California, Santa Barbara, Santa Barbara, CA 93106, USA; 2 Lead contact

## Abstract

The FANCD2-FANCI heterodimer contributes to DNA repair at interstrand crosslinks and sites of replication stress. This complex has been physically and mechanistically linked to double-strand break (DSB) repair, but its role in that process remains undefined. Here, we show that the FANCD2-FANCI heterodimer dynamically interacts with open chromatin regions, including transient DSB-induced open chromatin, where it can be stabilized through co-activation by the DNA repair kinase ATM and the Fanconi anemia core ubiquitin ligase. The loaded FANCD2-FANCI heterodimer stabilizes open chromatin and promotes resection and loading of RPA through increased association of BRCA1 and BLM. Chromatin-loaded FANCD2-FANCI has a second, distinct function promoting a G2 cell cycle arrest that is dependent on the ATR-CHK1-WEE1 axis. Our results support a two-step genome surveillance model in which FANCD2-FANCI monitors open chromatin sites and is stably loaded to coordinate DNA repair activities in response to signaling from a DNA repair kinase.

## In brief

Joyce et al. demonstrate that the FANCD2-FANCI heterodimer binds to open chromatin, including transiently accessible regions formed near double-strand breaks. Chromatin-loaded FANCD2-FANCI promotes DNA repair activities in the vicinity of double-strand breaks.

## Graphical Abstract

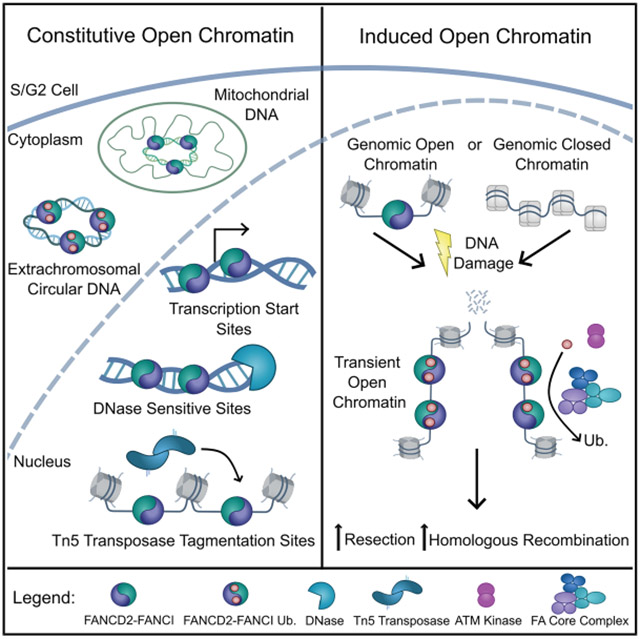

## INTRODUCTION

Fanconi anemia (FA) is a genetic disorder characterized by developmental abnormalities, bone marrow failure in early childhood, and an increased incidence of cancer, primarily hematologic and solid tumors.^1^ FA is caused by biallelic mutations in any of at least 22 genes that comprise the FA-BRCA pathway.^2^ The defining characteristic of an FA gene is that FA cells undergo chromosomal breakage upon exposure to DNA crosslinking agents like mitomycin C.^1^ This test illustrates that the FA-BRCA pathway functions to repair DNA, but hints that the FA-BRCA pathway may unite multiple different repair activities to deal with complex lesions like interstrand crosslinks (ICLs). It remains unclear whether the FA-BRCA pathway is activated by distinct DNA damage signals to perform lesion-specific repair or if there is a unifying model that describes the recruitment and activity of the pathway.

The key activation event in the FA-BRCA pathway is the clamping of the FANCD2-FANCI heterodimer onto DNA, facilitated by the monoubiquitination of FANCD2 and FANCI by the FA core ubiquitin ligase complex.^2^ Appearance of monoubiquitinated products on immunoblots or accumulation of ubiquitinated FANCD2-FANCI on chromatin is therefore used as a marker for underlying DNA damage.^3^ Another FA factor, FANCM, likely recruits the core complex to sites of DNA damage.^4,5^ The remaining genes in the FA-BRCA pathway include effector proteins that have well-defined roles in DNA repair, such as three RAD51 orthologs (FANCO/R/U), BRCA complex members (FANCD1/S/J/N), and nucleases (SLX4/XPF-FANCP/Q). The overall model that emerges from interactions within the FA-BRCA pathway indicates that DNA damage is sensed by the FA core complex, which clamps FANCD2-FANCI onto chromatin, wherein the heterodimer can facilitate downstream repair events. The function of individual FA genes reinforces the idea that it is a modular pathway that can be activated by multiple types of DNA damage and coordinate distinct repair activities.

The FA-BRCA pathway is best understood in its role in replication-dependent ICL repair. During this process, converging replication forks stall near the ICL, and FANCD2-FANCI associates with the lesion. The FA E3 ligase core complex monoubiquitinates the FANCD2-FANCI heterodimer at ICL-damaged chromatin. Subsequent recruitment of downstream factors excises crosslinks from DNA and rescues severed replication forks.^2^ This latter activity is similar to double-strand break (DSB) repair. DSB repair proteins belong to one of two pathways: end-joining (EJ) proteins that reseal the lesion or homologous recombination (HR) proteins that copy the sequence around the lesion from an intact template molecule.^6^ HR requires the conversion of the DSB into a recombination filament, utilizing the nuclease activity of the MRE11-RAD50-NBS1 (MRN) complex. Next, activation of long-range 5′ −3′ exonucleases BLM-DNA2 or EXO1 exposes single-stranded DNA, which RPA binds and protects from degradation by other nucleases. Finally, RAD51 replaces RPA, finalizing the formation of the recombination filament. BRCA1 and BRCA2 regulate events in this cascade, likely loading of RPA and RAD51, respectively. Outside of its canonical role in ICL repair, the FA-BRCA pathway has been shown to participate in fork protection and restart,^7,8^ colocalize with R-loops,^9^ maintain genome stability at chromosomal fragile sites,^10^ participate in the repair of UV-induced lesions,^11^ and localize to DSBs.^12,13^ It has been challenging to build lesion-specific models for the activation and function of the FA-BRCA pathway due to the diverse range of DNA substrates.

*In vitro* characterization of the FA-BRCA pathway suggests a lack of lesion-specific recruitment and activation. DNA binding experiments revealed that FANCD2-FANCI binds to many naked DNA substrates *in vitro*, with the highest affinity for branched DNA substrates that resemble stalled replication forks.^14^ Ubiquitination of FANCD2 by the FA core complex locks the closed FANCD2-FANCI heterodimer onto DNA substrates. High-resolution cryo-EM structures show unmodified as well as monoubiquitinated forms of the FANCD2-FANCI heterodimer bound to double-stranded DNA.^15-17^ These *in vitro* experiments describe a model in which the FANCD2-FANCI heterodimer binds to many DNA topologies and is then ubiquitinated by the FA core complex, undergoing a conformational change that increases binding affinity.

Our investigation into the recruitment and function of FANCD2-FANCI focused on its role at DSBs. Many FA-BRCA members, including FANCD2-FANCI, are genetically required for DSB repair, and depletion of these factors alters DSB repair outcomes.^12,18^ FANCD2-FANCI is recruited to DSBs after early DSB repair complexes, including the EJ complex DNA-PK, but before factors like RAD51 that participate in HR.^19^ These data support a model in which FA-BRCA members enable HR repair pathways. However, the precise role of the FANCD2-FANCI heterodimer at DSBs remains unclear. For instance, although FANCD2-FANCI associates with DSBs during repair, where we would expect to see FANCD2 bound to cut DNA, the majority of the DNA bound to FANCD2 does not end at the DSB, but in fact spans the DSB.^20^ This observation is consistent with FANCD2-FANCI bound to DSBs that have already been repaired, and therefore appears incompatible with the timing of its arrival at chromatin. We therefore set out to understand the substrate that FANCD2-FANCI binds to and its role at DSBs.

We isolated the DNA bound by the FANCD2-FANCI heterodimer to determine its DNA substrate in the context of DSB repair. We show that the FANCD2-FANCI heterodimer interacts dynamically with stable open chromatin sites in human cells and with transient open chromatin sites created by DNA repair events at DSBs. We use chromosome location- and cell cycle-specific assays to study FANCD2-FANCI at DSBs separate from their other replication or genome-maintenance functions. We find that FANCD2-FANCI is loaded onto chromatin in response to signaling at DSBs. Additionally, we find that FANCD2-FANCI is an integral part of the DNA resection machinery that assembles at DSBs, promoting resection and RPA loading. Finally, we show that FANCD2 alters cell cycle progression after DSB induction by promoting a G2 arrest via the ATR-CHK1-WEE1 pathway. Overall, our results support a model in which FANCD2-FANCI monitors open chromatin sites and regulates the accessibility of these sites to DNA repair proteins.

## RESULTS

### FANCD2-FANCI binds both single- and double-stranded DNA at DSBs, remaining bound throughout DNA resection

**Figure 1.**
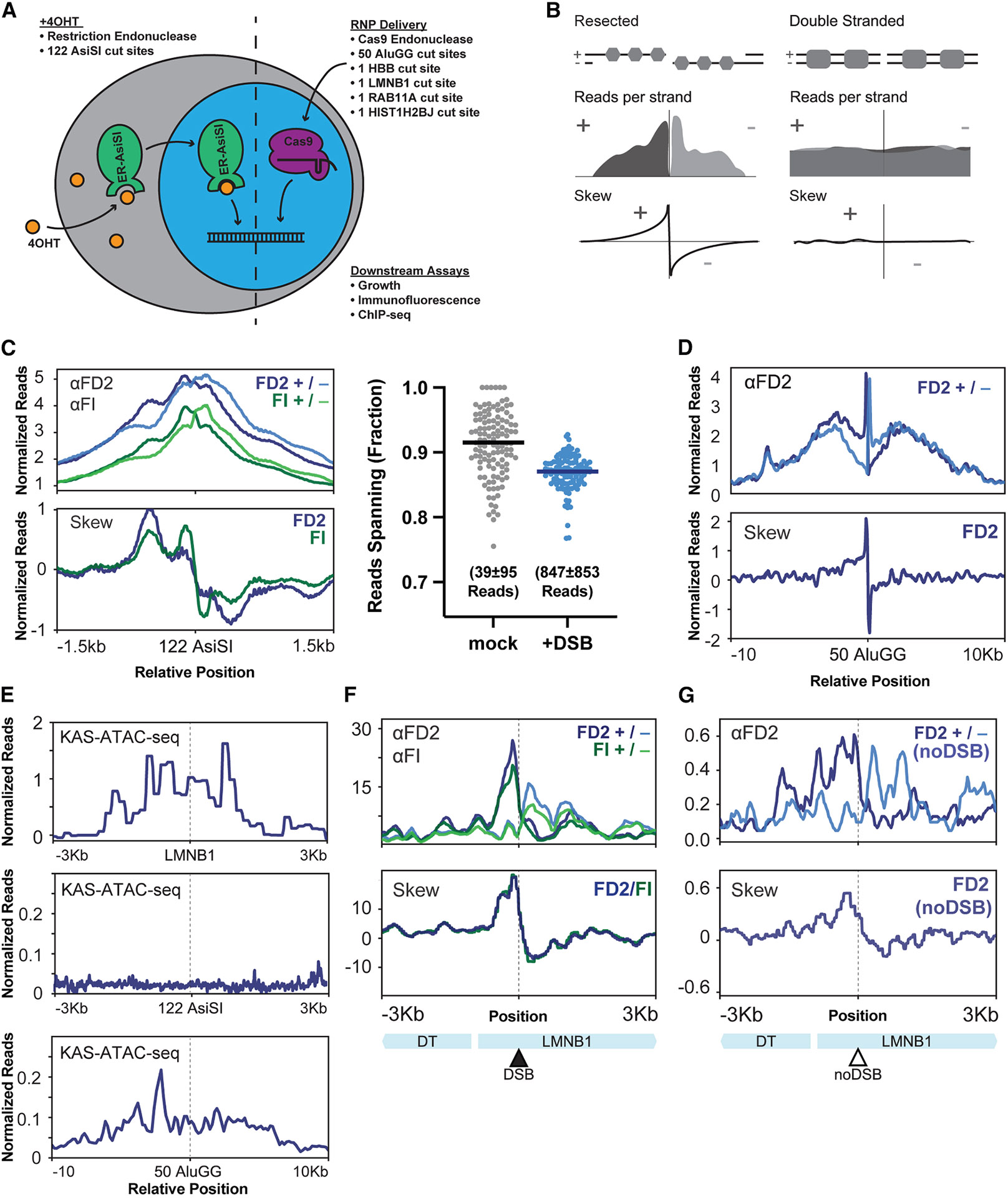
The FANCD2-FANCI heterodimer binds double- and single-stranded DNA at DSB sites (A) DSB induction schematic showing (1) addition of 300 nM 4OHT driving nuclear localization of the ER-AsiSI fusion protein and digestion of target sites or (2) electroporation of Cas9 and guide RNA targeting single or multiple genomic loci. (B) Stranded ChIP-seq schematic showing resected or double-stranded DNA, read profiles consistent with resected or double-stranded DNA, and skew profiles generated by subtracting reads mapping to the − strand from reads mapping to the + strand. (C) Stranded ChIP-seq data presented as reads mapping to distinct strands (top) or skew (bottom) at 122 AsiSI sites 4 h after DSB induction. Individual reads in FANCD2 ChIP-seq datasets spanning the DSB site were plotted as a frequency (right). Immunoprecipitations were performed using FANCD2 (FD2) or FANCI (FI) antibodies from DIvA-U2OS cells as indicated. Line plots are representative of *n* = 2 biological replicates and dot plots present all data from *n* = 2 replicates in each condition. (D) Stranded ChIP-seq data presented as reads mapping to distinct strands (top) or skew (bottom) at 50 AluGG sites 16 h after DSB induction. Immunoprecipitations were performed using FD2 antibody from U2OS cells. All plots are representative of *n* = 2 biological replicates. (E) Single-stranded DNA defined by KAS-ATAC-seq in GM12878 cells reproduced from PRJNA1103095 for the *LMNB1* site (top), 122 AsiSI sites (middle), or 50 AluGG sites (bottom). All plots are the average of *n* = 2 biological replicates. (F) Stranded ChIP-seq data presented as reads mapping to distinct strands (top) or skew (bottom) at the *LMNB1* cut site 16 h after DSB induction. Schematic shows the LMNB1 divergent transcript (DT) and LMNB1 transcript (LMNB1). Immunoprecipitations were performed using FD2 or FI antibodies from U2OS cells as indicated. Line plots are representative of *n* = 2 biological replicates. (G) Stranded ChIP-seq data presented as reads mapping to distinct strands (top) or skew (bottom) at the *LMNB1* locus in its natural (noDSB) context. Schematic shows the LMNB1 DT and LMNB1. Immunoprecipitations were performed using FD2 antibody from U2OS cells. Line plots are representative of *n* = 2 biological replicates.

Past studies have shown that FANCD2 is robustly recruited to DSBs, but the DNA bound to FANCD2 in these experiments was primarily uncut.^20^ This observation is difficult to reconcile with other results showing that disruption of FANCD2 alters DSB repair outcomes.^21^ To refine our understanding of FANCD2-FANCI interactions with damaged DNA, we performed inducible DSB assays at single or multiple loci. We monitored the induction of DSBs at 122 sites using the AsiSI endonuclease (5′ -GCGATCGC-3′), 50 sites using the AluGG guide RNA complexed with Cas9, or single sites using *HBB*, *LMNB1*, *RAB11A*, or *HIST1H2BJ* guide RNAs complexed with Cas9 (Figure 1A; Table S1).^22-24^ After DSB induction, we performed ChIP-seq experiments that preserved DNA strand information, and thus measured resected DNA (Figure 1B).

We observed that FANCD2-FANCI binds to DSBs 4 h after AsiSI induction (Figure 1C, top left plot). Consistent with previous results at single DSBs,^20^ most of the reads spanned the cut site, indicating that FANCD2-FANCI is mostly bound to intact DNA (Figure 1C, right plot). FANCD2-FANCI binding at AsiSI DSBs on average showed a preference for the 3′ strand on either side of the DSB, indicating that FANCD2-FANCI can bind single-stranded DNA. This trend can be seen by plotting read counts mapping to the + or − DNA strands, or alternatively by plotting read skews that report the excess of reads mapping to the + or − DNA strands on either side of the DSB (Figures 1B and 1C, bottom left plot). FANCD2 binds to AluGG DSB sites 16 h after DSB induction with a wider distribution and greater preference for the 3′ strand on either side of the DSB (Figure 1D). The width and strand preference of FANCD2-FANCI distributions at AsiSI and AluGG sites are consistent with the timing of DSB maturation events. For example, extensive 5′ to 3′ resection, which leaves single-stranded 3′ ends, would occur from 4 to 16 h after DSB formation. Overall, these results demonstrate that the FANCD2-FANCI heterodimer retains its binding during transitions at the DSB, binding both double-stranded DNA prior to resection activity and single-stranded DNA after resection activity.

Altogether, these data suggest that FANCD2 can remain bound throughout the repair process—even beyond resealing of the DSB. For example, DSB induction using a guide RNA that targets the N-terminus of the *HBB* gene causes a 9 base pair (bp) deletion of sequence adjacent to the DSB site. Immunoprecipitation of FANCD2 from cells with DSBs induced at *HBB* purifies DNA containing this 9-bp deletion (Figure S1A). This finding indicates that the FANCD2-FANCI heterodimer binds prior to DSB repair and remains bound throughout the repair process, including post-repair of a DSB.

To examine FANCD2-FANCI at a specific site, we targeted the *LMNB1* N-terminus. Analysis of existing kethoxal-assisted single-stranded DNA assay for transposase-accessible chromatin with sequencing (KAS-ATAC-seq) datasets,^25^ which labels denatured and single-stranded DNA in cells, revealed that the *LMNB1* locus, but not AsiSI or AluGG sites, contains accessible, single-stranded DNA prior to DSB induction (Figure 1E). Strandspecific ChIP-seq showed that FANCD2-FANCI binds asymmetrically to this single-stranded DNA both before and after cutting (Figures 1F and 1G), albeit with ~30-fold more signal following DSB induction. Asymmetric binding of FANCD2-FANCI to the uncut *LMNB1* locus is likely due to divergent transcription causing stably denatured DNA. Overall, these results demonstrate that FANCD2-FANCI binds to single-stranded DNA present before the DSB is introduced, as is the case with *LMNB1*, or to single-stranded DNA created by DSB resection, as is the case for AsiSI and AluGG DSBs. More broadly, this finding indicates that the FANCD2-FANCI heterodimer retains its binding distribution during transitions, either from double-stranded DNA to single-stranded DNA during resection, or from a pre-DSB to post-DSB chromatin state.

### FANCD2-FANCI is preloaded at open chromatin sites and further accumulates at DSB-induced open chromatin

**Figure 2.**
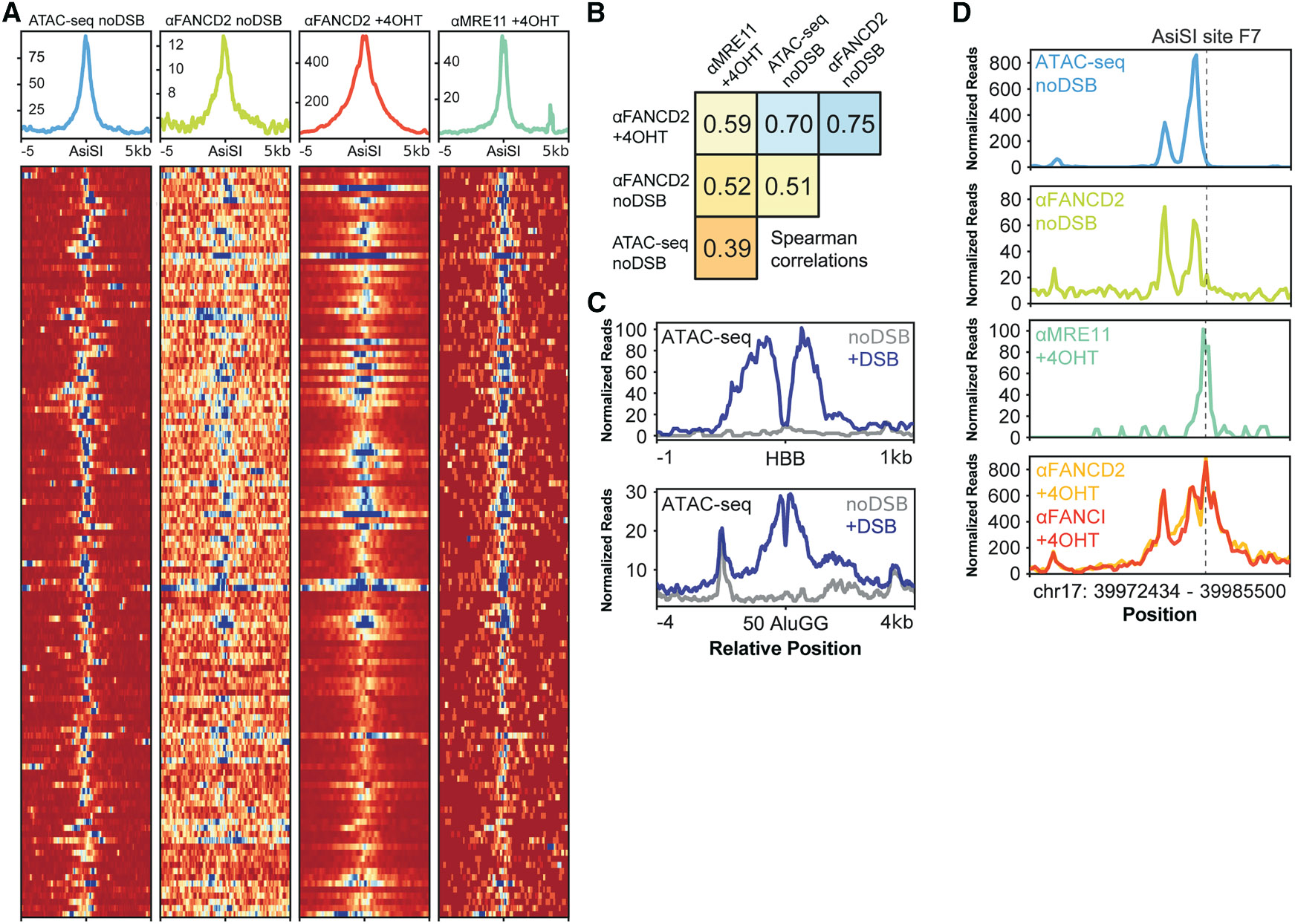
The FANCD2-FANCI heterodimer interacts dynamically with induced and stable open chromatin sites in human cells (A) ATAC-seq and ChIP-seq data presented as average and individual signal at 122 AsiSI sites either uncut (noDSB) or 4 h after DSB induction (+4OHT) and showing chromatin openness or repair factor recruitment. Immunoprecipitations were performed using MRE11 or FANCD2 antibodies from DIvA-U2OS cells as indicated. All plots are representative of *n* = 2 biological replicates. (B) Strong (≥0.7), moderate (≥0.5), and weak (<0.5) Spearman correlations between signal at all AsiSI recognition sites in the human genome (1,228 *in silico* predicted sites) generated by ATAC-seq without DSBs (noDSB), FANCD2 ChIP-seq without DSBs (noDSB), FANCD2 ChIP-seq with DSBs (+4OHT), and MRE11 ChIP-seq with DSBs (+4OHT). Data were generated from *n* = 2 biological replicates for each condition. (C) ATAC-seq signal at single (*HBB*) or multiple (50 AluGG) DSB sites without (noDSB) or 4 h after (+DSB) DSB induction in U2OS cells. Each panel shown is *n* = 1 biological replicate. (D) ATAC-seq and ChIP-seq data presented as signal at the F7 AsiSI site showing open chromatin via ATAC-seq (top), recruitment of FANCD2 via ChIP-seq without DSBs (second from top), recruitment of MRE11 via ChIP-seq with DSBs (second from bottom), and recruitment of FANCD2-FANCI via ChIP-seq with DSBs (bottom). Immunoprecipitations were performed using MRE11, FANCD2, or FANCI antibodies from DIvA-U2OS cells. Data shown are representative of *n* = 2 biological replicates

The pre-DSB distribution of FANCD2 at *LMNB1* led us to investigate whether chromatin-bound FANCD2-FANCI reflects a broad affinity for open chromatin. We compared FANCD2-FANCI binding before and after DSB induction at many sites in the genome using the AsiSI endonuclease. FANCD2 accumulates at AsiSI DSBs (Figure 2A, third plot), verified by MRE11 recruitment (Figure 2A, fourth plot). However, many of the AsiSI sites are also bound by FANCD2 prior to DSB induction (Figure 2A, second plot) and overlap with ATAC-seq-positive open chromatin (Figure 2A, first plot). Spearman correlations between these datasets indicate that ATAC-seq signal correlates with loading of FANCD2 in the absence of DSBs (Figure 2B). Furthermore, these correlations strongly predict the magnitude of FANCD2 association when DSBs are induced. We thus conclude that AsiSI sites in our dataset overlap with ATAC-seq-accessible chromatin and have underlying FANCD2-FANCI distributions, which are reinforced upon DSB induction.^26^

In contrast, Alu elements tend to occur in chromatin that is inaccessible to ATAC-seq. In the absence of DSBs, we see low levels of FANCD2 binding to 50 AluGG sites (Figure S1B). Upon DSB induction, FANCD2 robustly accumulates at these sites, binding both forward and reverse strands, as well as DNA that spans the cut site (Figures 1D and S1C). The FANCD2 distribution at AluGG DSB sites is biased toward binding to the 3′ -terminated strand, consistent with 5′ resection at the DSB (Figure 1D).

Overall, these results support a model in which FANCD2-FANCI binds DNA with a background, constitutive profile that follows the underlying chromatin accessibility. DSBs themselves cause transient ATAC-seq-positive open chromatin (Figure 2C),^23^ which allows FANCD2-FANCI loading. When FANCD2-FANCI is preloaded at open chromatin, the background binding pattern is preserved and amplified by the formation of an adjacent DSB. An example of this activity can be seen at AsiSI site F7, which has three open chromatin regions (Figure 2D, top plot) that associate with FANCD2 in the absence of DSBs, but do not overlap the consensus cut site (Figure 2D, second plot). DSB induction, verified by MRE11 recruitment (Figure 2D, third plot), increases FANCD2-FANCI signal at the DSB, preserves the non-DSB FANCD2-FANCI peaks, and increases overall signal by ~10-fold (Figure 2D, fourth plot). We therefore hypothesize that open chromatin—not DNA lesions—is the main substrate for FANCD2-FANCI.

To confirm that FANCD2-FANCI are bound together to the sites under investigation, we verified binding of both FANCD2 and FANCI across DSB sites and open chromatin using the re-ChIP sequential immunoprecipitation technique.^27^ Sequential immunoprecipitation of FANCD2-FANCD2, FANCI-FANCI, and FANCD2-FANCI produced robust signal at DSBs, DNase-positive open chromatin sites, and ATAC-seq-positive open chromatin sites (Figure S1D). Heterodimer (FANCD2-FANCI) signal was strongly correlated with homodimer (e.g., FANCD2-FANCD2) signal at each of these sites, which rules out homodimer binding to DSBs or open chromatin sites (Figure S1E). To confirm that binding of FANCD2 required FANCI, and vice versa, we depleted FANCD2 or FANCI using siRNA and monitored the association of the non-depleted partner at individual DSB sites. In all cases, depletion of FANCD2 or FANCI decreased the loading of the binding partner at DSBs (Figure S1F). While we did not see evidence of FANCD2-FANCD2 or FANCI-FANCI homodimer binding at the DSB and open chromatin sites studied in this manuscript, a genome-wide search uncovered putative FANCD2-FANCD2 homodimer binding sites that lacked FANCI signal (Figure S1G). Overall, these results confirm that the DSB and open chromatin sites presented in this manuscript are FANCD2-FANCI heterodimer binding sites.

### Binding of FANCD2-FANCI to open chromatin substrates requires the FA core complex

**Figure 3.**
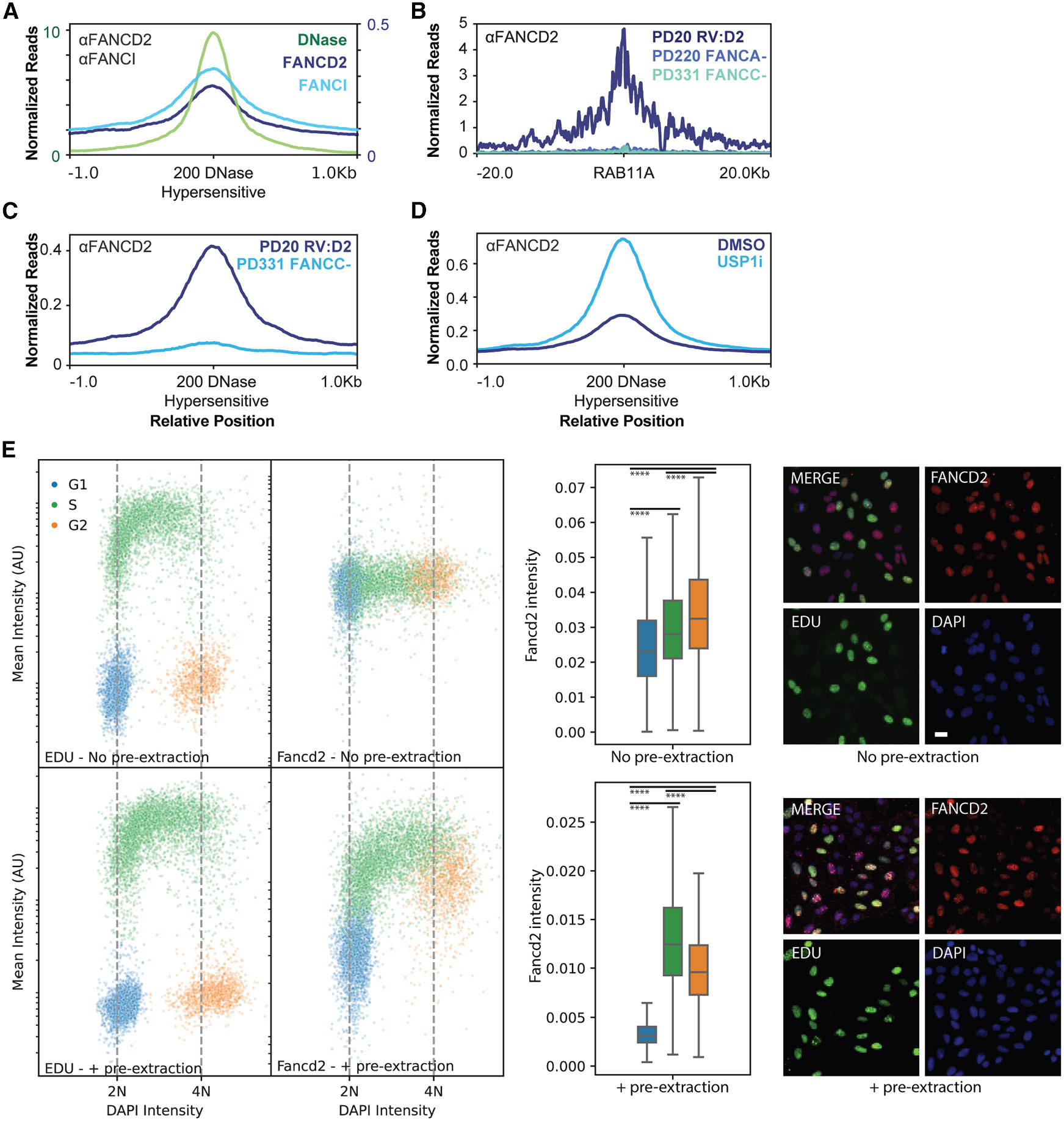
FANCD2-FANCI loading at open chromatin substrates requires the FA core complex and is cell cycle dependent (A) ChIP-seq profiles showing FANCD2 binding (U2OS cells) and FANCI binding (U2OS cells); DNase hypersensitivity profiles (MG63 cells) at 200 non-TSS DNase hypersensitive sites defined by the ENCODE consortium.^28^ Data shown are representative of *n* = 2 biological replicates. (B) ChIP-seq profiles showing FANCD2 binding 16 h after electroporation with Cas9 RNP targeting the *RAB11A* gene in patient-derived fibroblast cell lines expressing FANCD2 (PD20 RV:D2) or lacking the FA core components FANCA (PD220) or FANCC (PD331). Data shown are representative of *n* = 2 biological replicates. (C) ChIP-seq profiles showing FANCD2 binding to DNase hypersensitive sites in patient-derived fibroblast cell lines expressing FANCD2 (PD20 RV:D2) or lacking the FA core component FANCC (PD331). Data shown are representative of *n* = 2 biological replicates. (D) ChIP-seq profiles showing FANCD2 binding to DNase hypersensitive sites in U2OS cells with and without inhibition of the deubiquitinase USP1 by 30 μM ML323 for 4 h. Data shown are representative of *n* = 2 biological replicates. (E) QIBC immunofluorescence tracking EdU incorporation, DAPI incorporation, and FANCD2 chromatin binding in non-extracted (top row) or pre-extracted (bottom row) DIvA-U2OS cells. Data are presented as pseudo cell cycle plots (left), as box and whisker plots showing FANCD2 mean intensity as a function of cell cycle (middle), and as example IF images (right). Data shown are generated from at least *m* = 12 images and *n* = 2,000 cells, and the *p* values were derived from Shapiro-Wilk test, followed by non-parametric Kruskal-Wallis test, followed by Dunn test. ****p ≤ 0.0001. Scale bar represents 20 μm.

To further test whether FANCD2-FANCI binds open chromatin substrates, we compared both FANCD2 and FANCI binding to non-TSS DNase hypersensitive sites defined by the ENCODE consortium (Table S1) in osteosarcoma MG63 cells (Figure 3A).^28^ We found that FANCD2-FANCI is enriched at non-TSS DNase hypersensitive sites in the absence of DSBs. In addition to genomic open chromatin regions, our ChIP-seq datasets also revealed that FANCD2-FANCI binds to non-genomic open chromatin regions, including mitochondrial DNA^29^ and extrachromosomal DNA (Figure S2A).^30^

Consistent with past studies, we observed that FANCD2 is robustly recruited to DSBs in an FA-core-dependent manner (Figure 3B). Therefore, we sought to determine whether the binding of FANCD2-FANCI to open chromatin substrates also requires the FA core complex. In FANCC-null cell lines, FANCD2 abundance decreased at DNase hypersensitive sites, indicating that the binding of FANCD2-FANCI to these sites is stabilized by the FA core complex (Figure 3C). Additionally, we found that binding to plasmid-derived extrachromosomal DNA depends on the FA core (Figure S2B). Conversely, we tested the effect of inhibition of the FANCD2-FANCI deubiquitinase, USP1.^31^ Preventing de-ubiquitination of FANCD2-FANCI increased binding of FANCD2 at DNase hypersensitive sites (Figure 3D). USP1 inhibition also reduced recombination between a genomic DSB and genomic template provided by the DR-GFP system (Figure S2C).^32^ This observation is consistent with overloading of FANCD2-FANCI at non-DSB sites, thereby depleting the cellular pool of FANCD2-FANCI that is available for DNA repair. Together, these results support a model in which core-dependent FANCD2-FANCI loading occurs at open chromatin substrates.

### Binding of FANCD2-FANCI is cell cycle dependent

Quantitative image-based cytometry (QIBC)^33^ revealed that FANCD2 binds chromatin throughout the S and G2 phases but is excluded during G1 (Figure 3E, plus pre-extraction). This regulation is driven by chromatin loading rather than protein abundance, as FANCD2 protein abundance remains relatively stable throughout the cell cycle (Figure 3E, no pre-extraction). This finding contrasts with BRCA1, whose protein abundance decreases in G1 cells (Figure S2D, no pre-extraction). We further investigated FANCD2 binding in early versus late replicating regions, as defined by REPLIseq datasets.^34^ While replication timing did not influence localization to DSBs (Figure S2E, top plots), we observed a ~2-fold increase in FANCD2 signal at ATAC-seq-positive sites located in early replicating regions (Figure S2E, bottom plots). Since early replicating regions tend to overlap with open chromatin,^35^ we conclude that underlying accessibility influences FANCD2 recruitment. Overall, we favor a model in which FANCD2-FANCI samples open chromatin in the S and G2 phases of the cell cycle.

### ATM activation stabilizes FANCD2-FANCI at open chromatin sites during resection in the vicinity of DSBs

**Figure 4.**
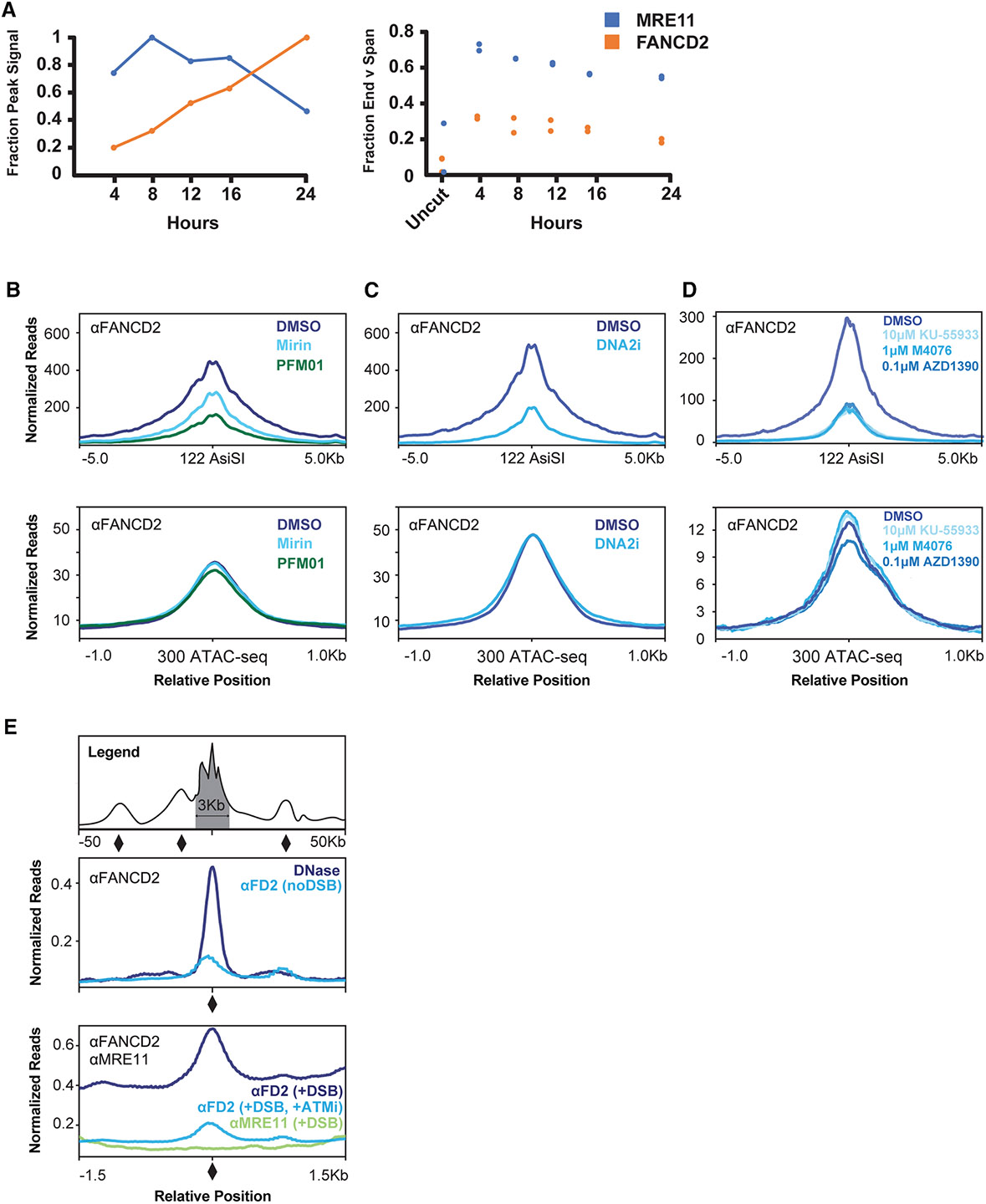
ATM activation stabilizes FANCD2-FANCI at DSBs and DSB-adjacent DNase hypersensitive sites (A) ChIP-seq summary data showing MRE11 and FANCD2 binding as a function of time after electroporation with Cas9 RNP targeting the *RAB11A* locus (left) and the fraction of reads ending at the DSB site in each condition (right). Data shown are generated from *n* = 2 biological replicates in U2OS cells. (B) ChIP-seq profiles showing FANCD2 binding to DSBs (top) or open chromatin sites (bottom) in untreated (DMSO), 100 μM mirin-treated, or 100 μM PFM01-treated conditions (4 h). Data shown are representative of *n* = 2 biological replicates in DIvA-U2OS cells simultaneously treated with 300 nM 4OHT for 4 h. (C) ChIP-seq profiles showing FANCD2 binding to DSBs (top) or open chromatin sites (bottom) in untreated (DMSO) or 2 μM NSC-105808-treated (DNA2i) conditions (4 h). Data shown are representative of *n* = 2 biological replicates in DIvA-U2OS cells simultaneously treated with 300 nM 4OHT for 4 h. (D) ChIP-seq profiles showing FANCD2 binding to DSBs (top) or open chromatin sites (bottom) in untreated (DMSO) or ATM inhibitor-treated (10 μM KU-55933, 1 μM M4076, or 0.1 μM AZD1390) conditions (4 h). Data shown are representative of *n* = 2 biological replicates in DIvA-U2OS cells simultaneously treated with 300 nM 4OHT for 4 h. (E) DNase hypersensitivity profiles (MG63 cells) at non-TSS DNase hypersensitive sites defined by the ENCODE consortium.^28^ ChIP-seq profiles showing FANCD2 binding (DIvA-U2OS cells) and MRE11 binding (DIvA-U2OS cells) at DSB-adjacent DNase-hypersensitive sites. These sites arise more than 1.5 kb but less than 50 kb upstream and downstream of 122 AsiSI cut sites (top) and average signal at these sites is presented without (middle; noDSB) or with (bottom; +DSB) DSB induction, and with ATM inhibitor treatment (10 μM KU-55933). Data shown are representative of *n* = 2 biological replicates in DIvA-U2OS cells. For DSB induction, cells were treated with 300 nM 4OHT (with or without ATMi) for 4 h.

To establish the timing of recruitment after DSB induction, we monitored the association of FANCD2 and MRE11 with Cas9-induced DSBs in the *RAB11A* promoter. We found that steadystate MRE11 association peaks approximately 8 h after DSB induction (Figure 4A, left plot), while FANCD2 continued to accumulate out to 24 h. The majority of the DNA bound to MRE11 ends at the cut site, indicating MRE11 is bound to unrepaired DNA (Figure 4A, right plot). FANCD2, on the other hand, is primarily bound to DNA that spans the cut site, consistent with a fraction of FANCD2 preloaded at open chromatin at the *RAB11A* locus.

Inhibition of MRE11 using the exonuclease domain inhibitor Mirin or the endonuclease domain inhibitor PFM01^36^ prevented FANCD2 binding, with endonuclease inhibition causing the greatest reduction in FANCD2 binding at AsiSI-induced DSBs (Figure 4B, top plot). Neither inhibitor affected FANCD2 binding to non-DSB ATAC-seq-positive open chromatin sites (Figure 4B, bottom plot). We conclude that FANCD2 accumulates after activation of the nuclease domains of MRE11, which contribute to short-range resection activity. MRE11 binding is also upstream of DNA2 and other long-range exonucleases. DNA2 inhibition with NSC-105808^37^ reduced FANCD2 association at DSBs (Figure 4C, top plot), but not at non-DSB ATAC-seq-positive open chromatin sites (Figure 4C, bottom plot). We attribute the reduction in FANCD2 loading at DSBs in the presence of PFM01, Mirin, and NSC-105808 as a reduction in open chromatin caused by reduced DSB resection.

Another role of MRE11 is the recruitment and activation of the DNA repair signaling kinase, ATM, at DSBs. Inhibition of ATM with KU-55933,^38,39^ M4076 (Lartesertib), or AZD1390, each verified by monitoring the phosphorylation status of CHK2 and γ-H2AX (Figure S3A), reduced FANCD2 association at DSBs (Figure 4D, top plot), but not at non-DSB ATAC-seq-positive open chromatin sites (Figure 4D, bottom plot). ATM inhibition also reduced FANCD2 association at DSB-adjacent open chromatin sites (Figure 4E). To investigate this, we compiled a list of DSB-adjacent open chromatin sites ranging from 1.5 kb to 50 kb upstream or downstream of DSBs (Figure 4E, indicated by diamonds on the representative first plot). These sites are DNase hypersensitive and load FANCD2 in the absence of DSBs (Figure 4E, second plot). Upon DSB induction, these sites accumulate additional FANCD2, but not MRE11, indicating that they are DSB-adjacent open chromatin sites but are not themselves DSBs (Figure 4E, third plot). ATM inhibition prevents additional FANCD2 accumulation at these sites (Figure 4E, compare third plot with second plot). We thus conclude that ATM inhibition reduces FANCD2 association with both DSBs and local, DSB-adjacent open chromatin.

Since DSB-adjacent open chromatin sites lie within the footprint of the ATM substrate, γ-H2AX, surrounding AsiSI cut sites,^40^ we speculate that ATM activity triggers FANCD2-FANCI ubiquitination and chromatin loading (Figures S3A and S3B). Comparing ChIP-seq signal in the presence or absence of ATM inhibition reveals that DSB induction drives a ~25-fold increase in binding, while ATM inhibition limits this to ~5-fold above background; thus, the majority of the signal represents newly recruited complexes (Figure S3C).

ATR, another DNA repair signaling kinase, promotes the association of FANCD2-FANCI with stalled replication forks.^41^ However, ATR kinase inhibition with AZ20 or AZD6738 (Ceralasertib) did not alter FANCD2 recruitment to DSBs 4 h after DSB induction in asynchronously dividing cells (Figure S3D, left plot), nor to non-DSB ATAC-seq-positive open chromatin sites (Figure S3D, right plot). A longer 24-h incubation with the ATR inhibitor AZ20 dramatically reduces FANCD2 binding at DSBs (Figure S3E, compare 24-h plots with 4-h plots for each individual DSB). As ATR depletion causes a G1 arrest (and FANCD2 does not bind chromatin in G1), any depletion of FANCD2 at this time point is likely secondary to cell cycle effects. Together, these results support a model where FANCD2-FANCI functions as a surveillance complex that samples open chromatin. Specific kinases, such as ATM at DSBs, license FANCD2-FANCI for chromatin loading, allowing distinct pathways to regulate FANCD2-FANCI in a lesion-specific manner.

### FANCD2-FANCI loading changes the recruitment of DNA repair proteins and chromatin accessibility in the vicinity of DSBs

**Figure 5.**
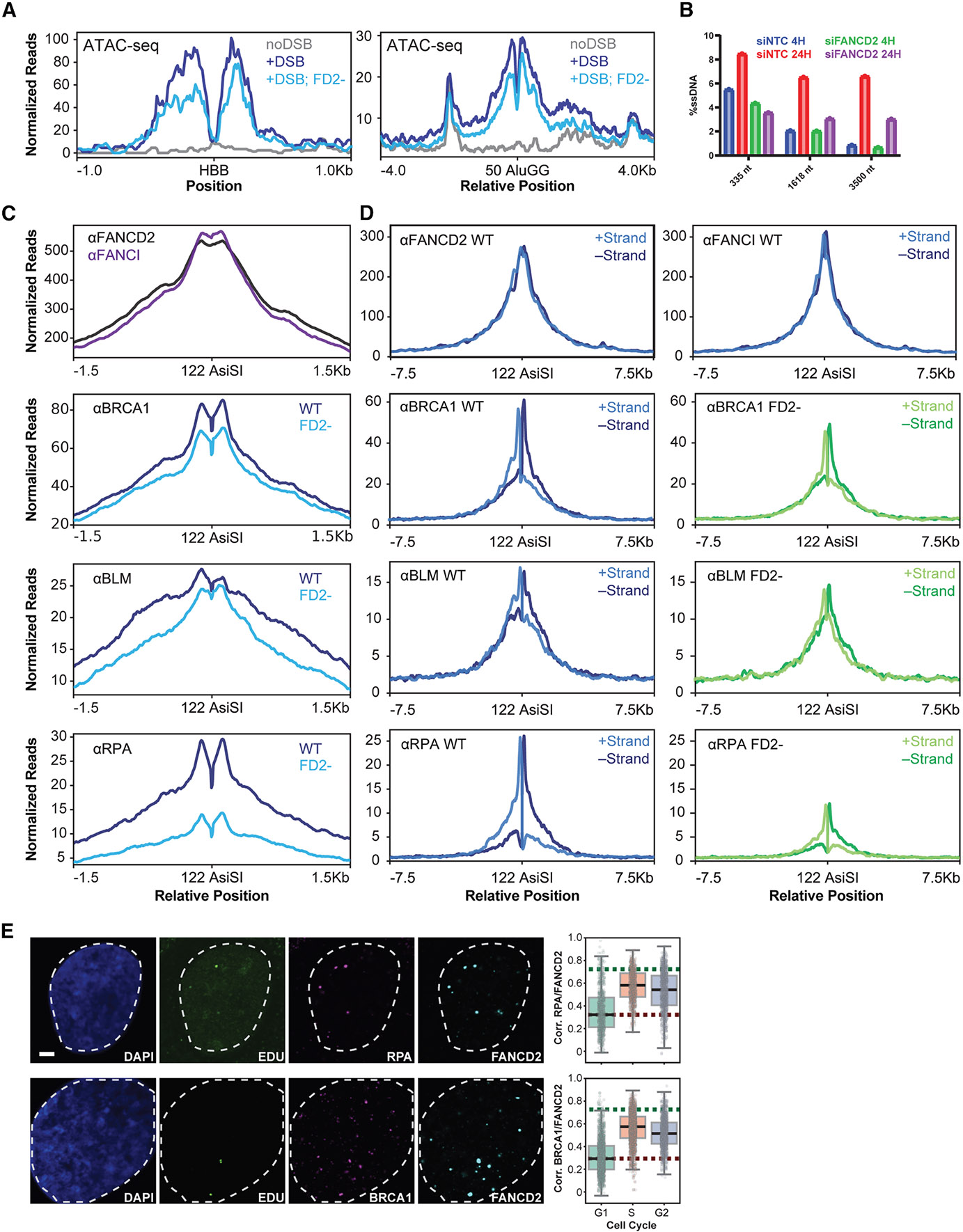
FANCD2-FANCI loading changes the recruitment of DNA repair proteins and chromatin factors in the vicinity of DSBs (A) ATAC-seq signal in U2OS cells at single (*HBB)* or multiple (50 AluGG) DSB sites in non-targeting control cells without DSBs (gray line; noDSB), in non-targeting control cells 4 h after DSB induction (purple line; +DSB), or FANCD2-depleted cells 4 h after DSB induction (blue line; +DSB; FD2−). Each panel shown is *n* = 1 biological replicate. (B) qPCR-based DNA resection assay showing the percentage of single-stranded DNA (%ssDNA) at three different distances from the DSB in non-targeting control (siNTC) and FANCD2-depleted (siFANCD2) DIvA-U2OS cells 4 and 24 h after DSB induction using 300 nM 4OHT. Data were generated from *n* = 3 technical replicates. (C) ChIP-seq profiles showing FANCD2-FANCI, BRCA1, BLM, and RPA signal at AsiSI DSBs in CRISPRi non-targeting control (WT) and FANCD2-depleted (FD2−) DIvA-AID-U2OS-ZIM3 cells 4 h after DSB induction using 300 nM 4OHT. Data shown are representative of *n* = 2 biological replicates. (D) Stranded ChIP-seq profiles showing FANCD2-FANCI, BRCA1, BLM, and RPA signal at AsiSI DSBs in CRISPRi non-targeting control (WT) and FANCD2-depleted (FD2−) DIvA-AID-U2OS-ZIM3 cells 4 h after DSB induction using 300 nM 4OHT. Data shown are representative of *n* = 2 biological replicates. (E) QIBC immunofluorescence tracking EdU incorporation, DAPI incorporation, and RPA or BRCA1 colocalization with chromatin-bound FANCD2 in pre-extracted DIvA-U2OS cells 24 h after DSB induction using 300 nM 4OHT. Example IF images are presented at left and Pearson correlations as a function of cell cycle are plotted at right as box and whisker plots. Data shown are generated from at least *m* = 12 images and *n* = 2,000 cells. Scale bar represents 2 μm.

To understand how FANCD2 binding alters DSB repair, we first monitored chromatin accessibility in FANCD2-depleted cells. FANCD2 depletion decreased ATAC-seq signal at both *HBB* and AluGG DSBs (Figure 5A), indicating that FANCD2 promotes chromatin openness at lesions. We next assessed DSB maturation using qPCR,^42^ which revealed that depletion of FANCD2 reduces the amount but not the extent of single-stranded DNA generated by resection at AsiSI DSBs (Figure 5B). Consistently, ChIP-seq and ChIP-qPCR showed reduced loading of resection factors BRCA1, BLM, and RPA in the absence of FANCD2 (Figures 5C and S4A). We note that FANCD2-FANCI, BRCA1, BLM, and RPA share the same distribution at AsiSI DSBs, which suggests a model in which these factors work together during DSB repair (Figure 5C). These factors preferentially bind the 3′ terminated strands surrounding the DSB, and FANCD2 depletion reduces their abundance, without altering strand preference (Figure 5D). Together, these results indicate that FANCD2 promotes the end-resection phase of DSB maturation.

To assess whether repair proteins are bound to the same DNA lesion as FANCD2, we monitored G2 colocalization using QIBC. We chose G2 because it is a point in the cell cycle when FANCD2 can be loaded onto chromatin (Figure 3E, plus extraction), but recruitment of DNA repair proteins to sites of replication stress is minimal. We found that FANCD2 and RPA, as well as FANCD2 and BRCA1, colocalize at DSBs in G2 (Figure 5E), but not in G1, when no FANCD2 binding occurs. Since the dominant effect of FANCD2 depletion is reduced resection and RPA abundance, we propose that FANCD2-FANCI works alongside BRCA1 and BLM to facilitate DNA resection and load RPA. Consistent with FANCD2 working with BRCA1 to promote RPA loading, co-depletion of FANCD2 and BRCA1 reduces recombination frequencies in the context of a genomic DSB and genomic template provided by the DR-GFP system (Figure S4B).

In contrast, FANCD2 depletion did not reduce MRE11 or BRCA2 abundance (Figure S4C). Both proteins displayed a narrow distribution distinct from FANCD2, but shared a similar preference for the 3′ terminated strand (Figure S4D). Altogether, since FANCD2 depletion does not decrease BRCA2 loading at DSBs, and since BRCA2 has a narrow distribution, similar to MRE11, we hypothesize that BRCA2 recruitment to DSBs is linked to events directly at the DSB and is distinct from the open chromatin binding shown by FANCD2-FANCI. Accordingly, co-depletion of FANCD2 and BRCA2 in the DR-GFP system has no additive effect compared to BRCA2 depletion alone (Figure S4B).

FANCD2 depletion causes a modest reduction of RAD51 loading at DSBs (Figure S4E). After DNA resection, RPA is replaced by RAD51, in a process facilitated by BRCA2.^6^ Further investigation of the strandedness of the DNA bound to RAD51 in unperturbed and FANCD2-depleted cells revealed that RAD51, like RPA, is bound primarily to the 3′ terminated strand (Figure S4F). We hypothesize that this result is likely the average of two opposing activities: decreased DNA resection and RPA abundance in FANCD2-depleted cells, but no decrease in the abundance of the RAD51-loading factor BRCA2. Using QIBC, we found that FANCD2 and RAD51 moderately colocalize at DSBs in G2 (Figure S4G) compared to γ-H2AX and phospho-ATM (Figure S4H). We therefore conclude that a subset of FANCD2 not only acts during DNA resection to alter DSB maturation but also remains bound throughout RAD51-mediated recombination.

Finally, FANCD2 depletion increased the recruitment of MRE11, BLM, BRCA1, and BRCA2 to non-DSB ATAC-seq-positive open chromatin sites (Figure S5A). This suggests that without FANCD2-FANCI stabilization, these regions may experience damage and mature into DSBs. As FANCD2-FANCI loading at these sites is ATM independent (Figure 4D, bottom plot), this suggests a broader role for FANCD2-FANCI in stabilizing open chromatin and maintaining overall genome fidelity.

### Chromatin-loaded FANCD2 promotes an ATR-dependent G2 cell cycle arrest

We showed that FANCD2-FANCI is bound to chromatin during the S and G2 cell cycle phases and excluded from chromatin in G1 (Figure 3E, plus extraction). Additionally, QIBC experiments revealed that DSB-dependent colocalization of FANCD2 with the HR repair factors BRCA1, RPA, and RAD51 occurred in G2 (Figures 5E and S4G). Therefore, we sought to understand whether chromatin-loaded FANCD2 itself could influence cell cycle progression.

**Figure 6.**
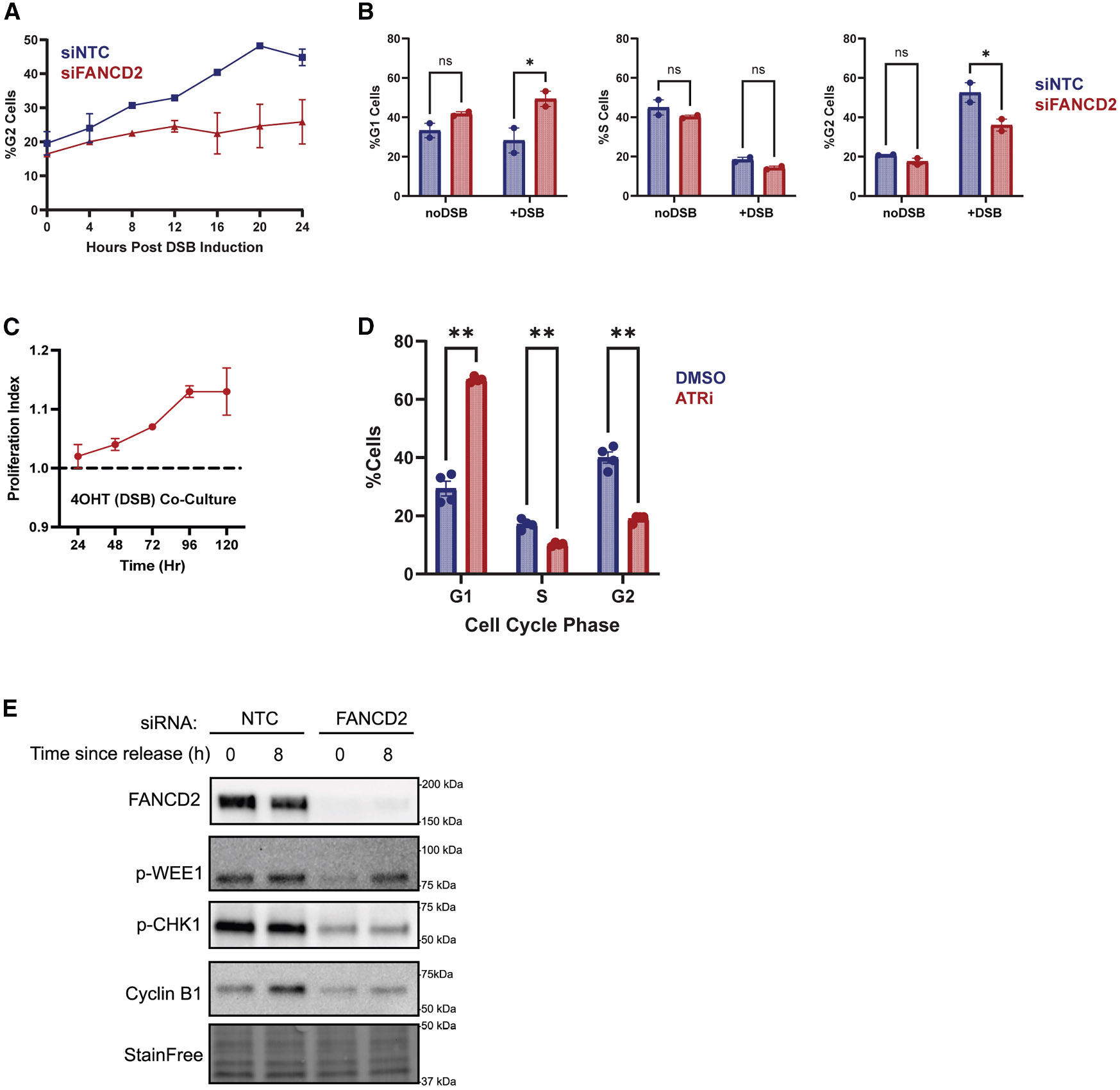
Chromatin-loaded FANCD2 promotes an ATR-dependent G2 arrest (A) Flow cytometry measurement of G2 cell cycle abundance in non-targeting control (siNTC) or FANCD2-depleted (siFANCD2) DIvA-U2OS cells at the indicated times after DSB induction using 300 nM 4OHT. Error bars indicate SEM. Data shown were generated from *n* = 2 biological replicates. (B) Flow cytometry cell cycle distributions of non-targeting control (siNTC) or FANCD2-depleted (siFANCD2) DIvA-U2OS cells either asynchronous (noDSB) or 24 h after DSB induction (+DSB). Left plot represents G1 phase, middle plot represents S phase, and right plot represents G2 phase. Error bars indicate SEM. Data shown were generated from *n* = 2 biological replicates, and the *p* values were derived from ordinary two-way ANOVA with full model, followed by Sidak’s multiple comparisons test, with a single pooled variance. *ns*, not significant; *p ≤ 0.05. (C) Co-culture experiments comparing growth rates of CRISPRi non-targeting control (WT) and FANCD2 knockdown (FD2−) DIvA-AID-U2OS-ZIM3 cells continuously grown in 300 nM 4OHT (to induce DSBs) relative to undamaged controls. Error bars indicate SEM. Data shown are generated from *n* = 2 biological replicates. (D) Cell cycle distributions measured by QIBC of untreated (DMSO) or ATR inhibitor-treated (1 μM AZ20) DIvA-U2OS cells 24 h after DSB induction. Error bars indicate SEM. Data shown are generated from at least *m* = 12 images and *n* = 2,000 cells, and the *p* values were derived from repeated-measure two-way ANOVA with the Geisser-Greenhouse correction, followed by Sidak’s multiple comparisons test, with individual variances computed for each comparison. **p ≤ 0.01. (E) Western blots showing phospho-CHK1 (p-CHK1) and phospho-WEE1 (p-WEE1) signal at indicated times after release from a double thymidine block into media containing 300 nM 4OHT in non-targeting control (siNTC) or FANCD2-depleted (siFANCD2) DIvA-U2OS cells. Data shown are representative of *n* = 3 biological replicates.

Previous research has shown that >20 DSBs causes a G2 cell cycle arrest through activation of the G2/M DNA damage checkpoint.^43^ Similarly, we found that induction of >100 DSBs using the AsiSI endonuclease causes asynchronously dividing cells to accumulate in G2 starting 4 h after DSB induction and peaking around 20 h after DSB induction (Figure 6A, siNTC). Depletion of FANCD2 reduces the frequency of G2 cells (Figure 6A, siFANCD2). We confirmed that FANCD2-depleted cells have a reduced G2 accumulation 24 h after DSB induction (Figure 6B, right plot, %G2 Cells). This reduction requires the FA core complex, as FANCA depletion also reduces the G2 arrest 24 h after DSB induction (Figure S6A). This observation of core dependency leads us to conclude that ubiquitinated FANCD2-FANCI clamped onto chromatin promotes an elongated G2 cell cycle arrest following DSB induction.

The lack of a G2 accumulation in FANCD2-depleted cells (Figure 6B, right plot, %G2 Cells) and the corresponding increase in G1 cells (Figure 6B, left plot, %G1 Cells) may indicate that FANCD2-depleted cells undergo mitosis despite the presence of DNA damage. We therefore hypothesized that FANCD2-depleted cells would divide more quickly in the presence of DNA damage than control cells. To test this hypothesis, we performed a co-culture assay using mock-treated and FANCD2-depleted cells. We found that FANCD2-depleted cells divided faster than the non-targeting controls when cultured in DSB-inducing conditions, relative to undamaged controls (Figure 6C). These data indicate that FANCD2-depleted cells not only continue to proliferate in the presence of DNA damage, but in fact proliferate more than control cells in the presence of DNA damaging agents. To validate this observation, we performed dye dilution flow cytometry experiments in which patient-derived FA fibroblasts were pulse labeled with a stable fluorescent dye. Dilution of this dye by proliferation allowed for cytometry-based tracking of cell proliferation. We observed that the FANCD2-null fibroblasts lost fluorescence more quickly than FANCD2-complemented cells in the presence of AluGG DSBs (Figure S6B).

Based on the results of both the co-culture and dye dilution experiments, we conclude that FANCD2-deficient cells have less of a cell cycle delay under DSB-inducing conditions than wild-type cells. We observed no change in the number of 53BP1 foci in G1 cells in FANCD2-deficient cultures compared to wild-type cultures after DSB induction (Figure S6C), indicating that the accelerated cell cycle of FANCD2-deficient cells in the presence of DNA damage does not cause an accumulation of unresolved lesions. FANCD2-deficient cells likely sustain this accelerated cell cycle because lesions are remodeled less (accelerating repair) and there are other DNA repair pathways, like mitotic DNA synthesis (MiDAS), which allow for repair of lesions before G1. Accelerated cell division in the presence of DNA-damaging agents appears to be a general feature of FA mutant cells, as shown by FANCD2-deficient cells proliferating faster than non-targeting control cells in the presence of the crosslinking agent, MMC, relative to undamaged controls (Figure S6D).

DSB-induced G2 cell cycle arrest is known to be driven by ATR kinase.^44^ Accordingly, inhibition of ATR using AZ20^45^ abrogates the G2 arrest in our cell lines (Figure 6D). Therefore, we explored links between FANCD2 and ATR signaling. RPA-coated single-stranded DNA is the main trigger for the recruitment and activation of ATR, with the help of three key ATR activator proteins (ATRIP, ETAA1, and TopBP1).^46^ Since ATR inhibition does not alter FANCD2 recruitment to DSBs 4 h after DSB induction (Figures S3D and S3E), we speculate that the reduction in RPA loading at DSBs in FANCD2-depleted cells limits the recruitment and activation of ATR. Consistent with a model in which FANCD2 acts upstream of ATR activation, we observed that depletion of ATR activators reduced recombination in the DR-GFP system at a magnitude equivalent to FANCD2 depletion (Figure S6E).

ATR is known to drive the G2 cell cycle arrest through the ATR-CHK1-WEE1 axis.^47^ Thus, one readout for ATR kinase activity is the accumulation of phosphorylated CHK1 and WEE1. We found that FANCD2-depleted cells accumulate less p-CHK1 and p-WEE1 when released from G1 synchronization in the presence of DSBs (Figures 6E and S6F). Phosphorylated WEE1 kinase inactivates CDK1 by phosphorylation, keeping the CDK-Cyclin B1 complex inactive and therefore maintaining a G2 arrest by preventing cells from entering mitosis.^48^ Thus, less p-WEE1 indicates that FANCD2-depleted cells do not arrest as strongly in G2 as control cells. Consistent with this result, immediately after release from the double thymidine block (0 h), both control cells and FANCD2-depleted cells have similar Cyclin B1 levels. After 8 h of DSB induction post release from the double thymidine block, control cells have accumulated high levels of Cyclin B1 as well as p-WEE1, maintaining the cells in G2 due to high p-WEE1 levels keeping CDK-Cyclin B1 inactive. In contrast, FANCD2-depleted cells have relatively low levels of Cyclin B1, which is consistent with the cells dividing, as Cyclin B1 is degraded during mitosis. We speculate that the reduction in RPA loading surrounding DSBs in FANCD2-deficient cells limits ATR recruitment, activation, and enforcement of a G2 cell cycle arrest, which is consistent with the reduction in the levels of p-CHK1, a key substrate of ATR kinase. Altogether, these results indicate that FANCD2-FANCI both promotes DSB repair activities and contributes to cell cycle regulation to ensure that repair is completed before mitosis.

## DISCUSSION

Here we report that FANCD2-FANCI functions as a chromatin surveillance complex that samples open chromatin regions and is subsequently stabilized at sites of DNA damage. FANCD2-FANCI efficiently localizes to transient open chromatin formed at DSBs in a manner that is dependent on the FA core complex and ATM kinase activity. Chromatin-loaded FANCD2-FANCI maintains chromatin accessibility and stabilizes BRCA1 and BLM at DSBs, thereby increasing DSB resection and RPA loading. FANCD2-FANCI also contributes to a G2 arrest in response to DNA damage. Together, these FANCD2-FANCI activities promote resection-based DSB repair outcomes (summarized in Figure S7).

The wide range of DNA substrates bound to FANCD2-FANCI supports persistent association of the complex with chromatin throughout DSB repair. We show that FANCD2 binds single-stranded and double-stranded DNA (Figures 1F and 1G) and identify FANCD2-bound DNA spanning the cut site with EJ deletion outcomes (Figure S1A).^49^ To reconcile these observations, we propose that FANCD2-FANCI interacts wherever chromatin accessibility increases between DNA-bound protein complexes. Signaling from a second complex—ATM in proximity to DSBs—triggers ubiquitination of FANCD2-FANCI and encirclement around DNA. This loaded form of FANCD2-FANCI acts as a scaffold for other repair factors and remains loaded throughout DNA repair processes. We reason that loaded FANCD2-FANCI is unable to diffuse past other protein complexes on DNA and therefore can be retained on single-stranded DNA generated by resection from an adjacent DSB. How and when FANCD2-FANCI is unloaded from DNA damage sites will require future study. Our results and those in the literature indicate that bulk unloading of FANCD2-FANCI occurs before anaphase, with remaining FANCD2 foci labeling sites of MiDAS^50^ or anaphase bridges.^51^

Reframing the FANCD2-FANCI heterodimer as a chromatin surveillance factor presents a unifying explanation for its diverse substrates and activities. There are many substrates that have been reported to interact with FANCD2-FANCI, including mitochondrial DNA,^29^ extrachromosomal DNA,^30^ viral DNA,^52^ transcriptional start sites,^53^ genomic DNA regulatory elements,^54^ and chronic fragile sites.^55^ All of these features are known or very likely to coincide with open chromatin. However, the chromatin context at these features has previously been treated as a consequence of the underlying biology, and not as the relevant parameter that is recognized by FANCD2-FANCI. Defining FANCD2-FANCI as an open chromatin surveillance factor addresses how a single complex can recognize and load onto so many diverse features. However, this framing also invites questions about when and how the heterodimer is stabilized at distinct sites and how loaded heterodimer contributes to downstream repair events.

We propose that FANCD2-FANCI associates with open chromatin and is stabilized in response to local regulation. This general model is compatible with FA pathway activation at specific lesions. At ICLs, for example, bidirectional replication forks stall in the vicinity of the ICL, fork reversal occurs, and then the FANCD2-FANCI heterodimer associates with the site of the lesion.^2^ FANCD2-FANCI is stabilized in the vicinity of the ICL by the FA core ubiquitin ligase. This core complex is likely recruited to stalled forks by FANCM,^56^ and ATR may phosphorylate FANCI to license FANCD2-FANCI chromatin loading.^57^ Our model predicts that the initial association of FANCD2-FANCI occurs because of an underlying affinity for open chromatin that may be produced during fork stalling. We further predict that FANCD2-FANCI interacts with open chromatin caused by other activities but is ultimately loaded through kinase activity and the FA core ubiquitin ligase. The specific details of these modifications may change at different open chromatin sites. For example, our data support that ATM licenses FANCD2-FANCI for loading at DSBs (Figure 4D) and that the FA core complex is present at DSBs and required for loading of FANCD2 at these lesions.^58^

A major prediction of this surveillance model is that large amounts of DNA damage could sequester FANCD2-FANCI away from normally open sites, thereby preventing its usual function in stabilizing these regions. Our data show that FANCD2-FANCI stabilizes open chromatin sites and prevents their progression into MRE11-bound DSBs (Figure S5A). Accordingly, any reduction—direct or indirect—in FA protein levels may elevate spontaneous lesions at open chromatin sites, increasing genomic stress. In this sense, FA proteins serve as both proactive sensors, preserving genome fidelity by preventing endogenous damage, and as reactive effectors, repairing exogenous DNA insults.

Finally, reframing the FANCD2-FANCI heterodimer as a genome surveillance complex suggests new models for the role of the FA pathway in human disease. In the disease FA, patient cells are sensitive to crosslinking agents and endogenous aldehydes.^1,59^ Our work provides a unifying model for this dual sensitivity and further predicts that any DNA damage that triggers chromatin opening can be misrepaired in FA-deficient cells. FANCD2-FANCI may also protect chronic open chromatin sites from genome instability. Together, we favor a model in which a general DNA repair defect—rather than an inability to repair a specific lesion—causes the disease FA. The DNA repair and genome protection activities of FANCD2-FANCI are also relevant to cancer biology. FA-deficient cells likely show decreased fidelity of DNA repair and increased genomic instability. Moreover, we show increased proliferation of FA-deficient cells in the presence of DNA damage, which may suggest that FA deficiency is itself oncogenic under some circumstances. From a clinical perspective, our work supports ongoing efforts to establish FA pathway status as a biomarker for cancer progression or synthetic lethal interactions with chemotherapeutics.^60^

### Limitations of the study

This work demonstrates that FANCD2-FANCI binds to open chromatin substrates. However, our approach does not fully capture the complex *in vivo* dynamics of FANCD2-FANCI, including changes in FANCD2-FANCI expression in different cell types and cell-cycle-dependent chromatin loading of the heterodimer. We therefore do not know how cells balance the available pool of FANCD2-FANCI with open chromatin substrates and what pathogenic consequences arise if equilibrium cannot be maintained. Furthermore, our definition of “open chromatin” is functional (based on DNase or transposase accessibility) and lacks a precise biophysical characterization. Finally, while we identified a specific role for ATM kinase in promoting FANCD2-FANCI loading at DSBs, this investigation was limited to a single damage context. Mechanisms that allow for different kinases to trigger loading of FANCD2-FANCI at other lesions (such as ICLs or stalled forks) were not explored. These limitations remain key areas for future study.

## STAR★METHODS

### EXPERIMENTAL MODEL AND STUDY PARTICIPANT DETAILS

DIvA AsiSI-ER-U2OS (female) cells and DIvA-AID AsiSI-ER-U2OS (female) cells (a gift from Gaëlle Legube’s Lab, Center for Integrative Biology, Toulouse, France), were grown in DMEM GlutaMAX (Cat#10569044; Gibco) that was supplemented with 10% fetal bovine serum (FBS) (Cat#S11550; R&D Systems) and 1% penicillin/streptomycin (P/S) (Cat#15140122; Gibco). Immortalized patient-derived fibroblast lines, PD20 (FANCD2 −/−) and PD20 RV:D2 (FANCD2 −/− complemented retrovirally with FANCD2 cDNA), PD220 (FANCA −/− ), PD331 (FANCC −/− ), and PD352 (FANCG −/− ) (a gift from Fanconi Anemia Research Materials, Oregon Health and Science University, Portland, Oregon, USA), were cultured in MEM alpha (Cat#12571071; Gibco) with 10% FBS and 1% P/S. K-562 (female) cells were obtained from ATCC, and cultured in RPMI 1640 (Cat#72400120; Gibco) with 10% FBS and 1% P/S. U2OS DR-GFP (female) cells (a gift from Jeremy Stark’s lab, City of Hope, Duarte, California, USA) were grown in DMEM with 10% FBS and 1% P/S. WT U2OS (female) cells obtained through ATCC were grown in DMEM with 10% FBS and 1% P/S. HEK293T (female) cells obtained through ATCC were grown in DMEM with 10% FBS and 1% P/S. DIvA AsiSI-ER-U2OS, PD20 RV:D2, and U2OS DR-GFP cell cultures were maintained in 1 μg/mL of puromycin dihydrochloride (Cat#A1113803; Gibco). DIvA-AID AsiSI-ER-U2OS cells were maintained in 800 μg/mL of Geneticin G418 sulfate (Cat#10131027; Gibco). All cell cultures were grown at 37°C and 5% CO2 in a humidified incubator. Cell lines were routinely tested for mycoplasma contamination using the MycoStrip Mycoplasma Detection Kit (Cat#rep-mys-50; InvivoGen).

### METHOD DETAILS

#### Molecular cloning

BFP-marked ZIM3 KRAB–dCas9 (pCR2122)^61^ and mCherry-marked gRNA vector in pIGI0056 with puromycin resistance (pCR2076) were used for CRISPRi. Guides were cloned into the BstXI-BlpI sites of pCR2076. Plasmids and protospacer sequences can be found in Table S1 (Plasmids tab).

#### Lentiviral packaging

Approximately 500,000 HEK293T cells were seeded per well in 6-well plates to achieve 50% confluency. The next day, 200 μL Opti-MEM Reduced Serum Media (Cat#31985070; Gibco) was incubated for 5 min with 12 μL of TransIT-LT1 Transfection Reagent (Cat#MIR2305; Mirus Bio) according to the manufacturer’s recommendations. Meanwhile, standard second-generation packaging plasmids (1.68 μg of delta VPR and 0.42 μg of VSV-G) were mixed together with 2.4 μg of the cargo plasmid (BFP-marked ZIM3 KRAB–dCas9 or BFP- or mCherry-marked gRNA vectors with puromycin resistance). Then, the two solutions were mixed together and allowed to incubate for 20 min, after which the entire solution was added dropwise over the HEK293T cells. The media was changed at 24H post transfection. Viral supernatants were harvested 48H–72H after transfection, centrifuged to remove any HEK293T cells, and filtered to remove additional debris. Cleared viral supernatants were either used fresh immediately in lentiviral transductions or flash frozen and stored at −80°C until use.

#### Lentiviral transduction

Approximately 200,000–250,000 DIvA-AID AsiSI-ER-U2OS cells were seeded per well in 6-well plates. The next day, the cells were transduced with viral supernatants. Viral titering was performed to optimize transduction conditions to ensure that <20% of the cells were infected. Cells were passively incubated with viral supernatants for 24H. After 24H, the media was changed three times (at 24H, 48H, 72H) to remove viral particles. For generation of BFP-marked ZIM3 KRAB–dCas9 DIvA-AID AsiSI-ER-U2OS cells, dilution cloning was performed and monoclones were selected by assessing BFP expression by flow cytometry (Attune NxT Flow Cytometer, Invitrogen). These BFP+ monoclones were then transduced with BFP or mCherry-marked gRNA vectors against FANCD2 or non-targeting controls. Cell cultures expressing additional BFP signal or mCherry signal were enriched through antibiotic selection by the addition of 1.5 μg/mL of puromycin dihydrochloride (Cat#A1113803; Gibco) starting 48H after transduction.

#### RNA interference (siRNA)

Approximately 200,000–250,000 WT U2OS, DIvA AsiSI-ER-U2OS, or U2OS DR-GFP cells were seeded per well in 6-well plates. The next day, the cells were transfected with 50 picomoles of each siRNA (designed against FANCD2, FANCI, FANCA, BRCA1, BRCA2, ATRIP, ETAA1, TopBP1, or a non-targeting control) using Lipofectamine 2000 Transfection Reagent (Cat#11668019; Invitrogen) according to manufacturer’s recommendation as follows: briefly, 5 μL of Lipofectamine 2000 was diluted into 150 μL of Opti-MEM Reduced Serum Media (Cat#31985070; Gibco) and allowed to incubate for 5 min. Meanwhile, 50 picomoles of each siRNA was diluted into 150 μL of Opti-MEM and allowed to incubate for 5 min. Then, the two solutions were mixed together and allowed to incubate for 20 min, after which the entire solution was added dropwise over the cells. The media was changed at 24H post transfection, and the cells were expanded at this time as well. Experiments (specifically DSB inductions) were conducted at 72H post transfection. For ChIP-scale experiments involving siRNA, this protocol was scaled up 10x, for both the number of cells and the amount of each reagent.

#### Cell cycle synchronization

Approximately 100,000 DIvA AsiSI-ER-U2OS cells were seeded per well in 6-well plates, 24H post siRNA treatment as described above. Later that same day, the cells were treated with 2 mM thymidine for 15 h. Cells were released using 3 washes of pre-warmed D-PBS, and the cells were replenished with fresh DMEM media for 9 h. Cells were then treated with 2 mM thymidine again for 15 h, before being released using 3 washes of pre-warmed D-PBS. Immediately post washing, cells were then replenished with fresh DMEM containing 300 nM 4-Hydroxytamoxifen (4-OHT) for 8 h to induce breaks. Samples were collected for western blotting at both the release (defined as 0H) and after break induction (defined as 8H of 4-OHT).

#### Western blotting

Cells in 6-well plates (approximately 250,000 cells per well at time of harvesting) were washed with D-PBS and then lysed directly in culture plates using 60 μL 2x Laemmli lysis sample buffer (containing 20% glycerol; 125 mM Tris–HCl, pH 6.8; 4% SDS; 0.02% bromophenol blue) supplemented with 5% 2-mercaptoethanol. After lysis, cells were collected by scraping and then transferred to 1.5-mL tubes. Next, samples were sonicated for 15 s on, 5 s off, for a “total on” time of 30 s at 50% amplitude (probe sonicator, Branson). Samples were then boiled at 95°C for 3 min, and finally centrifuged at max speed (21,100 x *g*) for 5 min. Samples were resolved with 4–20% SDS-PAGE gels (Cat#4568096; Bio-Rad), and protein levels were assessed utilizing Stain-Free total protein quantification. If protein levels differed, levels were normalized through relative pixel densitometry quantification of whole-lane Stain-Free signal. After SDS-PAGE resolution, proteins were transferred to 0.45 μm LF PVDF membranes by semi-dry transfer (Trans-Blot Turbo Transfer System, BioRad). Membranes were blocked for 1H with 3% Bovine Serum Albumin (Cat#BP1605-100; Fisher Scientific) in TBS containing 0.1% Tween 20 (TBS-T). Next, membranes were probed at 4°C overnight with primary antibodies, refer to Table S1 (WB Ab tab), in 3% BSA in TBS-T as described above. The next day, membranes were washed 3 times for 5 min each with TBS-T. Next, membranes were probed with species-specific secondary antibodies (goat anti-rabbit-IgG) conjugated to HRP in 3% BSA in TBS-T. After 1H of incubation with secondary antibodies, membranes were washed 3 times for 5 min each with TBS-T. Finally, chemiluminescence visualization was performed using either Pierce ECL 2 Western Blotting Substrate (Cat#PI80196; Thermo Scientific) or SuperSignal West Femto Maximum Sensitivity Substrate (Cat#34095; Thermo Scientific). Images were acquired on a ChemiDoc MP imaging system (BioRad).

#### Small molecule inhibition

DIvA AsiSI-ER-U2OS cells were treated with various small molecule inhibitors, with or without the addition of 300 nM 4-Hydroxytamoxifen (4-OHT) to the cell culture media to induce breaks. Typically, cells were treated for 4H for ChIP and for 24H for QIBC, unless otherwise specified. ML323, a USP1 inhibitor, was used at 30 μM. PFM01, an endonuclease inhibitor of MRE11, was used at 100 μM. Mirin, an exonuclease inhibitor of MRE11, was used at 100 μM. NSC-105808, a DNA2 inhibitor, was used at 2 μM. KU-55933, an ATM kinase inhibitor, was used at 10 μM. M4076 (Lartesertib), an ATM kinase inhibitor, was used at 1 μM. AZD1390, an ATM kinase inhibitor, was used at 100 nM. AZ20, an ATR kinase inhibitor, was used at 1 μM. AZD6738 (Ceralasertib), an ATR kinase inhibitor, was used at 1 μM. For recombination experiments using U2OS DR-GFP cells, small molecule inhibitors were added to the recovery medium following electroporation of the Cas9 RNP. After 16H of inhibitor treatment post electroporation (4D-Nucleofector X Unit and Core Unit, Lonza), cells were released from the inhibitors using 3 washes of pre-warmed D-PBS. Immediately post washing, cells were then replenished with fresh DMEM and allowed to recover until 96H post electroporation, at which time flow cytometry (Attune NxT Flow Cytometer, Invitrogen) was performed to measure recombination rates (refer to the flow cytometry section).

#### Cas9 and gRNA preparation

Cas9-NLS (*Streptococcus pyogenes*) aliquots were acquired from QB3 MacroLab from UC Berkeley. For gRNA production, single-stranded DNA oligos were annealed and PCR amplified to synthesize a DNA template containing a T7 promoter, 20bp protospacer (target-specific), 80bp handle (scaffold), and a polyA tail. The DNA template was transcribed into gRNA by a T7 RNA polymerase using the HiScribe T7 Quick High Yield RNA Synthesis Kit (Cat#E2050S; New England Biolabs), followed by removal of the DNA template by treatment with DNase I. Next, 5′ triphosphate groups were removed from the gRNA using an incubation with Shrimp Alkaline Phosphatase (Cat#M0371S; New England Biolabs). Finally, the gRNA was column purified, using either the RNeasy Mini Kit (Cat#74106; QIAGEN) for small-scale gRNA production or the Total RNA Purification Maxi Kit (Cat#26800; Norgen Biotek) for large-scale gRNA production. All gRNA protospacer sequences can be found in Table S1 (sgRNA tab).

#### Small-scale Cas9 RNP assembly and nucleofection

To measure recombination rates, 300 picomoles of *in vitro* transcribed SceGFP gRNA and 60 picomoles of purified Cas9-NLS (5:1 ratio of gRNA:Cas9) were allowed to complex for at least 10 min in 1x final RNP buffer (20 mM HEPES, pH 7.5; 200 mM KCl; 5 mM MgCl2; 5% glycerol; 1 mM TCEP). The total volume of RNP solution was kept at 5 μL, adjusted with nuclease-free water. Meanwhile, approximately 250,000 U2OS DR-GFP cells were lifted, pelleted by 3 min of centrifugation (500 x *g*), washed once with 1 mL D-PBS, and finally resuspended in 15 μL of Lonza Nucleofection Buffer SE (Cat#V4SC-1096; Lonza). Cells in SE buffer (15 μL) were mixed with the Cas9 RNP solution (5 μL), and the 20 μL total volume was transferred to cuvettes for electroporation using pulse code CM-104 for U2OS cells (4D-Nucleofector X Unit and Core Unit, Lonza). After electroporation, cells were left in the cuvettes for 5–10 min before being transferred to 6-well plates containing pre-warmed recovery media. For small molecule inhibitor experiments, the recovery media contained inhibitors for 16H of treatment post electroporation (refer to the small molecule inhibition section). Recombination rates were measured 96H post electroporation by flow cytometry (Attune NxT Flow Cytometer, Invitrogen).

#### Large-scale Cas9 RNP assembly and nucleofection

To generate DSB-induced cell pellets for ChIP, appropriately 2500 picomoles of *in vitro* transcribed gRNA and 500 picomoles of purified Cas9-NLS (5:1 ratio of gRNA:Cas9) were allowed to complex for at least 10 min in 1x final RNP buffer (20 mM HEPES, pH 7.5; 200 mM KCl; 5 mM MgCl2; 5% glycerol; 1 mM TCEP). The total volume of RNP solution was kept at 75 μL, adjusted with nuclease-free water. Meanwhile, approximately 5x10^6^ cells for U2OS cells or immortalized patient-derived fibroblast lines (or 20x10^6^ cells for K-562 cells) were lifted, pelleted by 3 min of centrifugation (500 x *g*), washed once with 10 mL D-PBS, and finally resuspended in the appropriate Lonza Nucleofection Buffer (see below). The volume of Lonza Nucleofection Buffer was kept as 50% of the total volume, taking into consideration the volume of the cell pellet (which was empirically determined for each cell type). Cells were electroporated using cell-specific pulse codes (see below). After electroporation, cells were left in the cuvettes for 5–10 min before being transferred to 15-cm plates containing pre-warmed recovery media. Lonza Nucleofection Buffer and pulse code for each cell line were as follows: U2OS cell lines were electroporated with Lonza Buffer SE and pulse code CM-104; immortalized patient-derived fibroblast lines were electroporated with Lonza Buffer P2 and pulse code CA-137; and K-562 cell lines were electroporated with Lonza Buffer SF and pulse code FF-120.

#### Co-culture proliferation assay

Non-targeting controls and FANCD2-depleted ZIM3 KRAB–dCas9 DIvA-AID AsiSI-ER-U2OS cells were labeled with either BFP or mCherry fluorescent markers (on the gRNA construct). Equal amounts of BFP+ cells and mCherry+ cells (approximately 50,000 cells each cell line) were seeded per well of 6-well plates after being mixed in the following 3 ways: (1) NTC D11 BFP and NTC D11 mCherry; (2) NTC D11 BFP and FANCD2 sgRNA1 mCherry; and (3) NTC D11 BFP and FANCD2 sgRNA2 mCherry. Mixed populations of the cells were either mock treated, treated with 300 nM 4-Hydroxytamoxifen (4-OHT) to induce DSBs, or treated with 50 ng/mL mitomycin C (MMC) to induce ICLs. Cells were harvested each day to assess the percentage of BFP and mCherry expression by flow cytometry (Attune NxT Flow Cytometer, Invitrogen). Each day the media was changed and fresh 300 nM 4-OHT or 50 ng/mL MMC was added to the cell culture media to continue to induce DSBs or ICLs, respectively. We quantified a proliferation index by taking the percentage of each cell line (NTC D11, FANCD2 sgRNA1, FANCD2 sgRNA2) in DNA damaging conditions (4-OHT or MMC) and normalizing to the percentage of each cell line in mock conditions. A proliferation index >1 would indicate that FANCD2-depleted cells with DSBs divide faster than FANCD2-depleted cells without DSBs, relative to co-cultured control cells.

#### Dye dilution flow cytometry experiments

Immortalized patient-derived fibroblast lines, PD20 (FANCD2 −/−) and PD20 RV:D2 (FANCD2 −/− complemented retrovirally with FANCD2 cDNA), were pulse-labeled with a membrane-permeable stable fluorescent dye using the CellTrace Violet Cell Proliferation Kit (Cat#C34557; Invitrogen). After labeling with 5 μM dye according to manufacturer’s recommendations, cells were either mock electroporated using a non-targeting gRNA or electroporated with a Cas9 RNP with AluGG gRNA (4D-Nucleofector X Unit and Core Unit, Lonza). Briefly, 300 picomoles of *in vitro* transcribed gRNA and 60 picomoles of purified Cas9-NLS (5:1 ratio of gRNA:-Cas9) were allowed to complex for at least 10 min in 1x final RNP buffer (20 mM HEPES, pH 7.5; 200 mM KCl; 5 mM MgCl2; 5% glycerol; 1 mM TCEP). Electroporation was performed using Lonza Nucleofection Buffer P2 and pulse code CA-137. Because the fluorescent dye is retained by the cells for several days after staining, cell proliferation after DSB induction was monitored by flow cytometry (Attune NxT Flow Cytometer, Invitrogen). Fluorescence intensity decreases as cell proliferation events occur.

#### Cell cycle analysis by flow cytometry

Approximately 30 min prior to each specified timepoint post DSB induction, DIvA AsiSI-ER-U2OS cells between 50% and 80% confluency were EdU pulsed for 30 min with 10 μM EdU. To perform fixation, cells were lifted, pelleted, and resuspended in a solution of D-PBS containing 4% formaldehyde for 10 min. After removing the fixative and performing a D-PBS wash, cells were then permeabilized on ice via a 15-min incubation in D-PBS containing 0.25% Triton X-100. Click chemistry was performed using a reaction mixture containing RB buffer (50 mM Tris–HCl, pH 7.5; 150 mM NaCl), copper sulfate (2 mM), fluorescent azide solution (5 μM), and freshly prepared sodium ascorbate (1 mg/mL). After a 30-min incubation in the dark at room temperature, cells were pelleted, washed once with D-PBS containing 0.25% Triton X-100, and then resuspended in 1 mL of D-PBS containing 1 μL of RNase A (10 mg/mL) and 5 μL of propidium iodide (1 mg/mL). Samples were incubated for 10 min at 37°C and then were analyzed by flow cytometry (Attune NxT Flow Cytometer, Invitrogen).

#### Flow cytometry

For experiments that used flow cytometry (recombination rate, co-culture, dye dilution, and cell cycle analysis), cells were analyzed via flow cytometry (Attune NxT Flow Cytometer, Invitrogen) and the software FlowJo v10.7.1. Cells were first gated for viability using forward scatter (FSC) and side scatter (SSC), using FSC-A vs. SSC-A. Viable cells were then gated for singlets using SSC-H vs. SSC-A. For recombination rate in U2OS DR-GFP cells, GFP+ cells were gated from singlets using FSC-A vs. BL1-H. For co-culture experiments, BFP+ cells and mCherry+ cells were gated using VL1-H and YL2-H, respectively. For dye dilution experiments to track cell proliferation, CellTrace Violet staining was analyzed using VL1-H. For cell cycle analysis, G1 and G2 cells were separated by propidium iodide staining and gated using YL2-H. S phase cells were separated from G1 and G2 cells by click chemistry using an Alexa Fluor 647 dye after EdU incorporation, and then gated using RL1-H.

#### Immunofluorescence microscopy and QIBC

Cells were grown in glass-bottom 96-well plates (Cat#P96-1.5H-N; Cellvis) with cell confluency between 50% and 80% at the time of fixation. Just prior to fixation, cells were EdU pulsed for 30 min with 10 μM EdU. When appropriate, pre-extraction was performed before fixation using ice-cold CSK buffer (10 mM PIPES–KOH, pH 6.8; 100 mM NaCl; 10% v/v sucrose; 1 mM EGTA; 1 mM MgCl2) containing 0.25% Triton X-100 for 2 min. Fixation was performed using a solution of D-PBS containing 4% formaldehyde for 10 min.

Cells were permeabilized with 0.25% Triton X-100 in D-PBS for 10 min. Click chemistry was performed using a reaction mixture containing RB buffer (50 mM Tris–HCl, pH 7.5; 150 mM NaCl), copper sulfate (2 mM), fluorescent azide solution (5 μM), and freshly prepared sodium ascorbate (1 mg/mL). After a 30-min incubation in the dark at room temperature, the click reaction buffer was removed. Next, cells were washed once with blocking buffer (D-PBS containing 0.1% Tween 20 and 3% BSA) and then incubated in blocking buffer for 30 min to 1 h at room temperature.

Primary antibodies, refer to Table S1 (IF QIBC Ab tab), were diluted in blocking buffer and incubated overnight at 4°C with rocking. Cells were then washed three times with PBS-T (D-PBS with 0.1% Tween 20) for 2 min each wash. Secondary antibodies and DAPI were diluted in blocking buffer and incubated for 1 h at room temperature in the dark. After incubation, three additional washes were performed with PBS-T. Finally, samples were stored in PBS and protected from light until imaging.

Images were acquired on a spinning disc confocal microscope (Nikon Ti2-E inverted microscope with a Yokogawa CSU-W1 spinning disk unit and an ORCA-Fusion BT sCMOS camera) equipped with a plan apo 10× Lambda D objective (NA = 0.45) for QIBC and a 100X 1.49 NA oil immersion objective for close-up imaging. To avoid vignetting on image borders, only the center of the image, representing one-quarter of the original field, was saved. Per well, 9 to 16 images were taken using 1 × 1 binning.

For QIBC, image analysis was performed using CellProfiler (v4.2.6, Broad Institute). Background levels for each channel were determined and subtracted in CellProfiler by assessing the 10% intensity value for each image. Subtraction was performed using the ImageMath module. Intensity measurements and foci quantification were performed exclusively within nuclei, delineated using a mask generated from DAPI nuclear staining. Foci levels were enhanced before thresholding using the EnhanceOrSuppress Features module using a feature size of 5. Quantification and plotting were performed in Python (v3.13.5) using Pandas (v2.3.3) and Seaborn (v0.13.2).

#### Chromatin immunoprecipitation

5x10^6^-20x10^6^ cells were prepared for ChIP by harvesting and resuspending in 15 mL of the appropriate room temperature cell culture media without serum. Cells were then crosslinked in suspension by the addition of 1 mL of 16% formaldehyde (Cat#28908; Thermo Scientific) for 10 min at room temperature with rotation (final concentration of 1% formaldehyde). The reaction was quenched with the addition of 0.85 mL 2.5 M glycine for 5 min under the same conditions (final concentration of 0.125 M glycine). Cells were washed 3 times with ice-cold D-PBS, each time being centrifuged at 1,200 x *g* for 3 min. Finally, the fixed cell pellets either proceeded immediately to lysis or were flash frozen and stored at −80°C until use.

Buffer solutions for cell lysis and nuclear fractionation were prepared ahead of time and placed on ice. Cell pellets were resuspended in 1 mL LB1 lysis buffer (50 mM HEPES–KOH, pH 7.5; 140 mM NaCl; 1 mM EDTA; 10% Glycerol; 0.5% IGEPAL CA-630; 0.25% Triton X-100) supplemented with protease inhibitors (Cat#PI78429; Fisher Scientific) and incubated in this buffer for 10 min at 4°C with rotation. Cells were then centrifuged at 2,000 x *g* at 4°C for 3 min, and after aspirating the supernatant, the new pellets were resuspended in 1 mL LB2 lysis buffer (10 mM Tris–HCl, pH 8.0; 200 mM NaCl; 1 mM EDTA; 0.5 mM EGTA) supplemented with protease inhibitors. Cells were incubated in this buffer for 5 min at 4°C with rotation, centrifuged at 2,000 x *g* at 4°C for 3 min, and after aspirating the supernatant, the new pellets were resuspended in 0.5 mL LB3 lysis buffer (10 mM Tris–HCl, pH 8.0; 100 mM NaCl; 1 mM EDTA; 0.5 mM EGTA; 0.1% Na–Deoxycholate; 0.5% N-Lauroylsarcosine) supplemented with protease inhibitors. Chromatin was sheared in the LB3 lysis buffer using a Qsonica Cup Horn Sonicator, 15 s on, 45 s off, for a “total on” time of 6 min at 25–50% amplitude, empirically determined based on the cell type and number of cells. 25 μL of each sonicated sample was collected for a sonication size check. The final shear size ranged between 200 and 600 bp. Samples were centrifuged at 21,100 x *g* for 10 min at 4°C to clear the lysates. Immunoprecipitations were carried out using the cleared sonicated lysate, which was brought up to 1 mL final volume with additional LB3 lysis buffer and 100 μL of 10% Triton X-100 (final concentration 1%). After saving 5% of each sample as input, each IP sample received 50 μL of Dynabeads protein A magnetic beads (Cat#10001D; Invitrogen) or 50 μL of Dynabeads protein G magnetic beads (Cat#10003D; Invitrogen) that were pre-conjugated to 3–10 μg of appropriate antibody, refer to Table S1 (ChIP Ab tab), prior to addition to each IP sample. Samples were incubated overnight at 4°C with rotation.

The next day, samples were washed 5 times with ice-cold ChIP-Wash buffer (50 mM HEPES–KOH, pH 7.5; 500 mM LiCl; 1 mM EDTA; 1% IGEPAL CA-630; 0.7% Na–Deoxycholate). Then, a single wash was performed with ice-cold TBS (20 mM Tris–HCl, pH 8.0; 150 mM NaCl). Samples were then eluted twice on a heated shaker at 65°C and 1200 rpm for 15 min each elution using 60 μL ChIP elution buffer (0.1 M NaHCO3; 1% SDS) per elution. To reverse DNA-protein crosslinks, samples were incubated overnight at 65°C on a heated shaker at 1200 rpm using 200 mM final concentration NaCl, alongside treatment with 2 μL RNase A(10 mg/mL). The next day, samples were incubated with 2 μL Proteinase K (20 mg/mL) on a heated shaker at 65°C and 1200 rpm for 1H. Finally, samples were purified using the MinElute PCR Purification Kit (Cat#28004; QIAGEN). ChIP samples were checked for efficacy using qPCR (refer to the qPCR section).

#### qPCR

Quantitative PCR was utilized as an efficacy check for ChIP experiments. 3 μL of template ChIP DNA was added to a master mix of 1.9 μL nuclease-free water, 0.05 μL of 100 μM forward primer, 0.05 μL of 100 μM reverse primer, and 5 μL of 2x SsoAdvanced Universal SYBR Green Supermix (Cat#1725271; BioRad). 45 cycles of a two-step qPCR were run and melting curve analysis was performed from 65°C to 95°C in increments of 0.5°C over 5 s per increment. Samples that amplified above 35 cycles were determined to be non-usable. All oligo sequences can be found in Table S1 (Oligos tab).

#### Resection assay

We utilized a previously established qPCR assay to measure the amount of single-stranded DNA after DSB induction in DIvA AsiSI-ER-U2OS cells.^42^ All oligo sequences can be found in Table S1 (Oligos tab).

#### Stranded library prep

Stranded library preps were performed on select ChIP samples using the xGen^™^ ssDNA & Low-Input DNA Library Preparation Kit (Cat#10009817; Integrated DNA Technologies) according to manufacturer’s recommendations.

#### ATAC-seq

ATAC-seq was accomplished by sending approximately 100,000 cells to Novogene (Novogene, Beijing) for tagmentation and high throughput sequencing.

#### High throughput sequencing

Non-stranded DNA library preparations and high throughput sequencing were performed using the PE150 sequencing chemistry (Illumina) on the NovaSeq platform (Novogene, Beijing).

#### ChIP-seq data analysis

Reads were quality and adaptor trimmed using fastp (v0.23.2), aligned to the hg38 genome (or to custom genome derivatives such as template plasmid chromosomes) using Bowtie 2 (v2.5.3), and deduplicated using picard. Reads mapping to the ENCODE blacklist sites (hg38-blacklist.v2.bed) were removed. The resulting bam files were processed in experiment-level batches using the deepTools (v3.5.1) packages multiBamSummary –scalingFactors and bamCoverage –scaleFactor to produce TMM normalized bigwig files. Where appropriate, reads mapping to the + (99, 147) and − (83, 163) strand were filtered into separate bam files using SAMtools (v1.20) flags.

TMM normalized replicate bigwig files were combined using bigWigCompare –operation mean. Where appropriate, combined replicate bigwigs were subtracted from combined control bigwigs using bigWigCompare –operation subtract.

Replicate bigwig files were visualized using computeMatrix and either plotHeatmap or plotProfile using custom BED files reproduced in Table S1.

#### ATAC-seq data analysis

ATAC-seq data analysis was performed as described for ChIP-seq, except that reads mapping to the mitochondria and reads that did not map in a proper pair, were removed.

### QUANTIFICATION AND STATISTICAL ANALYSIS

Statistical analyses were performed using GraphPad Prism v10.6.1. General details of each statistical comparison are provided in the figure legends. All statistical tests, sample numbers, and measurements are defined for each figure in Table S1 (Statistics tab). Significance definitions are as follows: *ns*, not significant; *p ≤ 0.05; **p ≤ 0.01; ***p ≤ 0.001; ****p ≤ 0.0001.

## Supplementary Material

1

2

Supplemental information can be found online at https://doi.org/10.1016/j.celrep.2025.116830.

## Figures and Tables

**Table:** KEY RESOURCES TABLE

REAGENT or RESOURCE	SOURCE	IDENTIFIER
Antibodies		
Anti-FANCD2 (Rabbit)	Abcam	Cat# ab221932; RRID: AB_3717344
Anti-FANCI (Rabbit)	Invitrogen	Cat# PA5-59014; RRID: AB_2643072
Anti-MRE11 (Rabbit)	Novus Biologicals	Cat# NB100-142; RRID: AB_350079
Anti-Histone-H4 (Rabbit)	Abcam	Cat# ab7311; RRID: AB_305837
Anti-BRCA1 (Mouse)	Santa Cruz Biotech	Cat# sc-6954; RRID: AB_626761
Anti-BLM (Rabbit)	Abcam	Cat# ab2179; RRID: AB_2290411
Anti-RPA (Mouse)	Millipore Sigma	Cat# MABE285; RRID: AB_11213221
Anti-BRCA2 (Rabbit)	Bethyl Laboratories	Cat# A300-005A; RRID: AB_2067772
Anti-RAD51 (Mouse)	Novus Biologicals	Cat# NB100-148; RRID: AB_10002131
Anti-phospho-CHK1-ser345 (Rabbit)	Cell Signal Tech	Cat# 2348; RRID: AB_331212
Anti-phospho-CHK2-thr68 (Rabbit)	Cell Signal Tech	Cat# 2661, RRID: AB_331479
Anti-phospho-H2A.X-ser139 (Rabbit)	Cell Signal Tech	Cat# 9718; RRID: AB_2118009
Anti-phospho-WEE1-ser642 (Rabbit)	Cell Signal Tech	Cat# 4910; RRID: AB_2215870
Anti-CyclinB1 (Rabbit)	Cell Signal Tech	Cat# 12231; RRID: AB_2783553
Anti-Rabbit-IgG (H + L) HRP Conjugate (Goat)	BioRad	Cat# 170–6515; RRID: AB_11125142
Anti-53BP1 (Mouse)	Millipore Sigma	Cat# MAB3802; RRID: AB_11212586
Anti-phospho-ATM-ser1981 (Mouse)	Cell Signal Tech	Cat# 4526; RRID: AB_2062663
Anti-pH3-ser10 (Mouse)	Cell Signal Tech	Cat# 9706; RRID: AB_331748
Goat anti-Mouse IgG (H + L) Highly Cross-Adsorbed Secondary Antibody, Alexa Fluor^™^ Plus 594 (Goat)	Invitrogen	Cat# A32742; RRID: AB_2762825
Anti-Rabbit IgG (H + L) Highly Cross-Adsorbed Secondary Antibody, Alexa Fluor^™^ Plus 647 (Goat)	Invitrogen	Cat# A32733; RRID: AB_2866492
Bacterial and virus strains		
*Escherichia coli* strain DH5α	–	N/A
*Escherichia coli* strain STBL3	–	N/A
Chemicals, peptides, and recombinant proteins		
Cas9-NLS (*Streptococcus pyogenes*)	UC Berkeley QB3 MacroLab	N/A
(Z)-4-Hydroxytamoxifen (4-OHT)	Millipore Sigma	Cat# H7904
ML323 (USP1 DUB Inhibitor)	SelleckChem	Cat# S7529
PFM01 (MRE11 Endonuclease Domain Inhibitor)	Millipore Sigma	Cat# SML1735
Mirin (MRE11 Exonuclease Domain Inhibitor)	Millipore Sigma	Cat# M9948
NSC-105808 (DNA2 Inhibitor)	National Cancer Institute (NCI) Developmental Therapeutics Program (DTP)	N/A
KU-55933 (ATM Kinase Inhibitor)	SelleckChem	Cat# S1092
M4076 (Lartesertib) (ATM Kinase Inhibitor)	SelleckChem	Cat# E1057
AZD1390 (ATM Kinase Inhibitor)	SelleckChem	Cat# S8680
AZ20 (ATR Kinase Inhibitor)	SelleckChem	Cat# S7050
AZD6738 (Ceralasertib) (ATR Kinase Inhibitor)	SelleckChem	Cat# S7693
Mitomycin C (MMC)	Millipore Sigma	Cat# M5353
Thymidine	Millipore Sigma	Cat# T1895
5-Ethynyl-2′-deoxyuridine (EdU)	Vector Laboratories	Cat# CCT-1149
AZDye 488 Azide	Vector Laboratories	Cat# CCT-1275
4′,6-Diamidino-2-Phenylindole (DAPI) Nucleic Acid Stain	ThermoFisher	Cat# D1306
Propidium Iodide	Millipore Sigma	Cat# P4170
DMEM GlutaMAX	Gibco	Cat# 10569044
Fetal Bovine Serum (FBS)	R&D Systems	Cat# S11550
Penicillin/Streptomycin (P/S)	Gibco	Cat# 15140122
MEM Alpha	Gibco	Cat# 12571071
RPMI 1640	Gibco	Cat# 72400120
Puromycin Dihydrochloride	Gibco	Cat# A1113803
Geneticin G418 Sulfate	Gibco	Cat# 10131027
Opti-MEM Reduced Serum Media	Gibco	Cat# 31985070
Bovine Serum Albumin	Fisher Scientific	Cat# BP1605-100
Pierce ECL 2 Western Blotting Substrate	Thermo Scientific	Cat# PI80196
SuperSignal West Femto Maximum Sensitivity Substrate	Thermo Scientific	Cat# 34095
Shrimp Alkaline Phosphatase (rSAP)	New England Biolabs	Cat# M0371
16% Formaldehyde (w/v), Methanol-free	Thermo Scientific	Cat# 28908
Halt Protease Inhibitor Cocktail (100X)	Fisher Scientific	Cat# PI78429
Proteinase K from *Tritirachium album*	Millipore Sigma	Cat# P6556
Monarch RNase A	New England Biolabs	Cat# T3018L
Critical commercial assays		
TransIT-LT1 Transfection Reagent	Mirus Bio	Cat# MIR2305
Lipofectamine 2000 Transfection Reagent	Invitrogen	Cat# 11668019
4–20% Mini-PROTEAN TGX Stain-Free Protein Gels	Bio-Rad	Cat# 4568096
SE Cell Line 96-well Nucleofector Kit	Lonza	Cat# V4SC-1096
SE Cell Line 4D-Nucleofector X Kit L	Lonza	Cat# V4XC-1024
SF Cell Line 4D-Nucleofector X Kit L	Lonza	Cat# V4XC-2024
P2 Primary Cell 4D-Nucleofector X Kit L	Lonza	Cat# V4XP-2024
Dynabeads Protein A for Immunoprecipitation	Invitrogen	Cat# 10001D
Dynabeads Protein G for Immunoprecipitation	Invitrogen	Cat# 10003D
SsoAdvanced Universal SYBR Green Supermix	Bio-Rad	Cat# 1725271
HiScribe^®^ T7 Quick High Yield RNA Synthesis Kit	New England Biolabs	Cat# E2050S
Total RNA Purification Maxi Kit	Norgen BioTek	Cat# 26800
RNeasy Mini Kit	Qiagen	Cat# 74106
MinElute PCR Purification Kit	Qiagen	Cat# 28004
xGen^™^ ssDNA & Low-Input DNA Library Preparation Kit	Integrated DNA Technologies	Cat# 10009817
CellTrace Violet Cell Proliferation Kit	Invitrogen	Cat# C34557
Click-It EdU Alexa Fluor 488 Imaging Kit	Invitrogen	Cat# C10337
Click-It EdU Alexa Fluor 647 Imaging Kit	Invitrogen	Cat# C10340
Deposited data		
DNase Hypersensitivity Dataset	ENCODE	SRA# ENCFF790EMB
γ-H2AX-seq Dataset	ENCODE	SRA# PRJEB21297
KAS-ATAC-seq Dataset	ENCODE	SRA# PRJNA1103095
REPLIseq Dataset	ENCODE	SRA# PRJNA397123
ChIP-seq and ATAC-seq	This Paper	SRA# PRJNA1249941
Experimental models: Cell lines		
U2OS	ATCC	HTB-96
K-562	ATCC	CCL-243
HEK293T	ATCC	CRL-3216
DIvA AsiSI-ER-U2OS	Gaëlle Legube Lab	N/A
DIvA-AID AsiSI-ER-U2OS	Gaëlle Legube Lab	N/A
U2OS DR-GFP	Jeremy Stark Lab	N/A
Patient-derived Fibroblast Cell Line PD20 RV:D2	Fanconi Anemia Research Materials, Oregon Health and Science University	N/A
Patient-derived Fibroblast Cell Line PD20 FANCD2−/−	Fanconi Anemia Research Materials, Oregon Health and Science University	N/A
Patient-derived Fibroblast Cell Line PD220 FANCA−/−	Fanconi Anemia Research Materials, Oregon Health and Science University	N/A
Patient-derived Fibroblast Cell Line PD331 FANCC−/−	Fanconi Anemia Research Materials, Oregon Health and Science University	N/A
Patient-derived Fibroblast Cell Line PD352 FANCG−/−	Fanconi Anemia Research Materials, Oregon Health and Science University	N/A
Oligonucleotides		
Silencer Select Negative Control No. 1 siRNA	Invitrogen	Cat# 4390843
Silencer Select siRNA Targeting FANCD2	Invitrogen	Cat# 4392420; siRNA ID #s4989
Silencer Select siRNA Targeting FANCI	Invitrogen	Cat# 4392420; siRNA ID #s226829
Silencer Select siRNA Targeting FANCA	Invitrogen	Cat# 4392420; siRNA ID #s162
Silencer Select siRNA Targeting BRCA1	Invitrogen	Cat# 4390824; siRNA ID #s457
Silencer Select siRNA Targeting BRCA2	Invitrogen	Cat# 4392420; siRNA ID #s2083
siRNA Targeting ATRIP	Millipore Sigma	Cat# NM_032166 siRNA ID SASI_Hs02_00359409
siRNA Targeting ETAA1	Millipore Sigma	Cat# NM_019002; siRNA ID SASI_Hs02_00352513
siRNA Targeting TOPBP1	Millipore Sigma	Cat# NM_007027; siRNA ID SASI_Hs02_00342794
Recombinant DNA		
AICSDP-52: HIST1H2BJ-mEGFP (pCR2020)	Allen Institute for Cell Science Plasmids	Addgene Plasmid #109121
AICSDP-10:LMNB1-mEGFP (pCR2002)	Allen Institute for Cell Science Plasmids	Addgene Plasmid #87422
pLX303-ZIM3-KRAB-dCas9 (BFP) (pCR2122)	Alerasool et al.^61^	Addgene Plasmid #154472
NTC D11 sgRNA (BFP-puro) (pCR1323)	Richardson et al.^12^	Addgene Plasmid #111125
NTC D11 sgRNA (mCherry-puro) (pCR2076)	This paper	N/A
NTC 1200 sgRNA (mCherry-puro) (pCR2145)	This paper	N/A
FANCD2 sgRNA1 (mCherry-puro) (pCR2146)	This paper	N/A
FANCD2 sgRNA2 (mCherry-puro) (pCR2147)	This paper	N/A
Software and algorithms		
Fastp	Chen et al.^62^	v0.23.2
Bowtie 2	Langmead et al.^63^	v2.5.3
SAMtools	Li et al.^64^	v1.20
deepTools	Ramírez et al.^65^	v3.5.1
Python	https://www.python.org/	v3.13.5
Seaborn	–	v0.13.2
Pandas	–	v2.3.3
CellProfiler	Broad Institute; https://cellprofiler.org/	v4.2.6
NIS-Elements	Nikon; https://www.microscope.healthcare.nikon.com/products/software/nis-elements	v5.42.01
GraphPad Prism	https://www.graphpad.com/	v10.6.1
FlowJo	https://www.flowjo.com/	v10.7.1

## Highlights

- The FANCD2-FANCI heterodimer associates with accessible chromatin, including DSBs
- Loading of FANCD2-FANCI is due to cell cycle, FA core complex, and ATM kinase
- Depletion of FANCD2 reduces resection activity and the loading of resection factors
- FANCD2 promotes an ATR-dependent G2 cell cycle arrest following DSB induction
