# Supplementary material for: The FANCD2-FANCI heterodimer coordinates chromatin openness and cell cycle progression throughout DNA double-strand break repair
Source: Cell Rep. Author manuscript; Available in PMC 2026 Feb 25. (PMC12934461; doi:10.1016/j.celrep.2025.116830)
Supplement: 1 [file NIHMS2142894-supplement-1.pdf]

Cell Reports, Volume *45*

Supplemental information

The FANCD2-FANCI heterodimer coordinates chromatin openness and cell cycle progression throughout DNA double-strand break repair

Christine M. Joyce, Julien Bacal, Soham P. Chowdhury, Andrew N. Brown, Amy K. Wang, Carmen Cruz, Kameron Bains, Zachary N. Rodriguez, Nathan J. McCormick, Yaara Tzadikario, Katherine U. Tavasoli, Brooke M. Gardner, and Chris D. Richardson

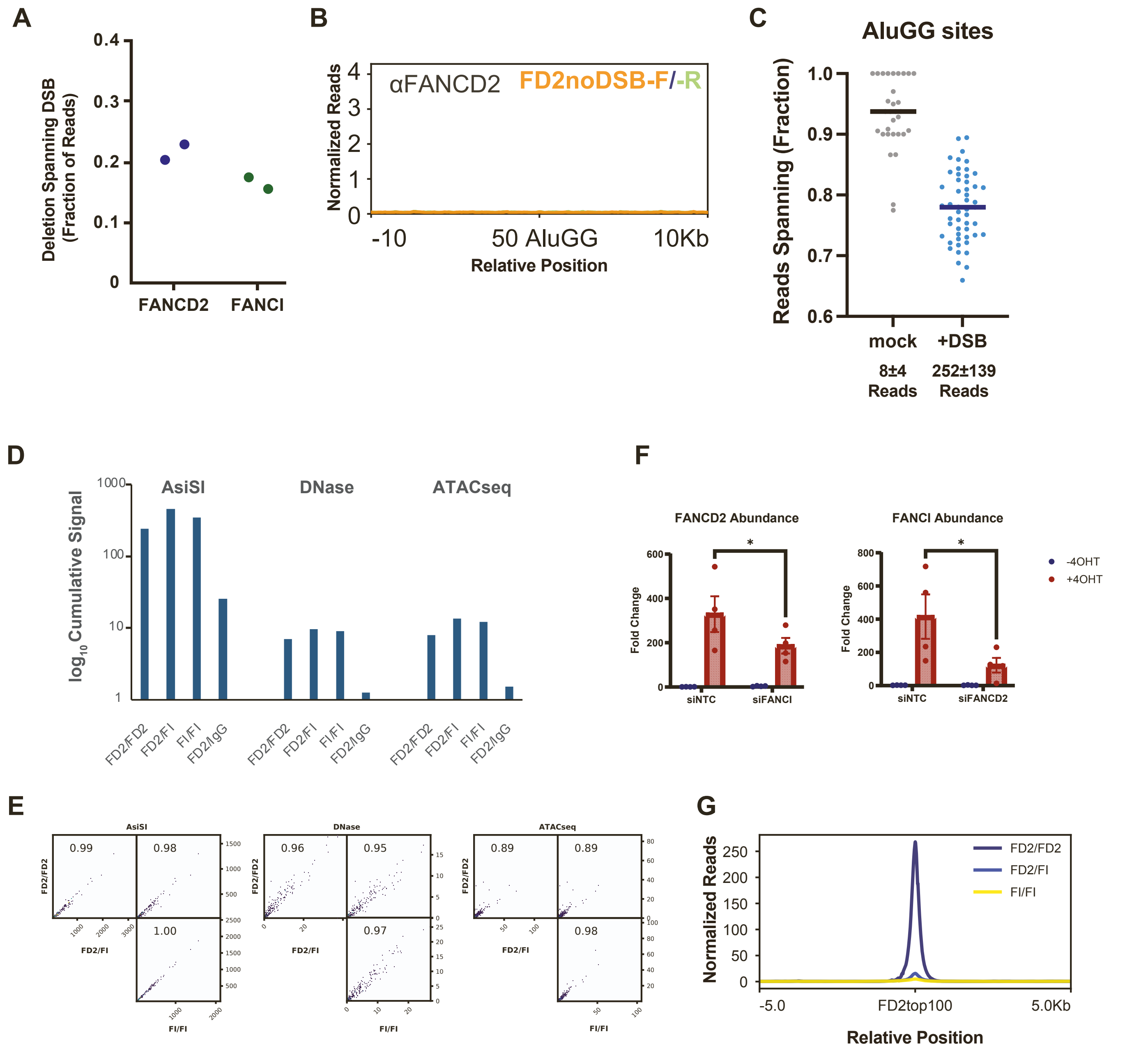

Figure S1:

A. Frequency of ChIP reads within a 200bp window surrounding the HBB DSB that encode a 9bp deletion that spans the site of the Cas9 DSB in FANCD2 (blue) or FANCI (green) ChIP-seq experiments. Data from n=2 biological replicates is plotted.

B. Stranded ChIP-seq data presented as reads mapping to distinct strands at 50 AluGG sites in their natural context (no DSB). Immunoprecipitations were performed using a FANCD2 antibody. All plots are representative of n=2 biological replicates.

C. Frequency of individual reads in the FANCD2 ChIP dataset from Figure 1D spanning the AluGG DSB sites. All data from n=2 biological replicates is plotted.

D. Summary of re-ChIP signal in a -2.5kb to +2.5kb window surrounding 122 AsiSI sites, 200 DNase hypersensitive sites, or 300 ATAC-seq sites for the indicated sequential immunoprecipitations (FD2/FD2, FD2/FI, FI/FI, and FD2/IgG). Immunoprecipitations were performed using FANCD2, FANCI, or IgG antibodies from DIvA-U2OS cells treated with 300 nM 4-OHT for 4 hours.

E. Pairwise correlations between signal in FD2/FD2 vs FD2/I, FI/FI vs FD2/FI, and FD2/FD2 vs FI/FI re-ChIP samples at AsiSI, DNase hypersensitive, or ATAC-seq sites.

F. ChIP-qPCR signal monitoring FANCD2 abundance (left plot) or FANCI abundance (right plot) to a single AsiSI cut site (chr1:88992917) with or without DSB induction in control (siNTC), FANCD2-depleted (siFANCD2), or FANCI-depleted (siFANCI) DIvA-U2OS. For DSB induction, cells were treated with 300 nM 4-OHT for 4 hours. Immunoprecipitations were performed using FANCD2 or FANCI antibodies as indicated. All data from n=2 biological replicates with n=2 technical replicates is plotted, and the p values were derived from ordinary two-way ANOVA with full model, followed by uncorrected Fisher's LSD, with a single pooled variance. $*p \leq 0.05$.

G. Average profiles of FD2/FD2, FD2/FI, and FI/FI sequential immunoprecipitations at putative FANCD2 homodimer sites.

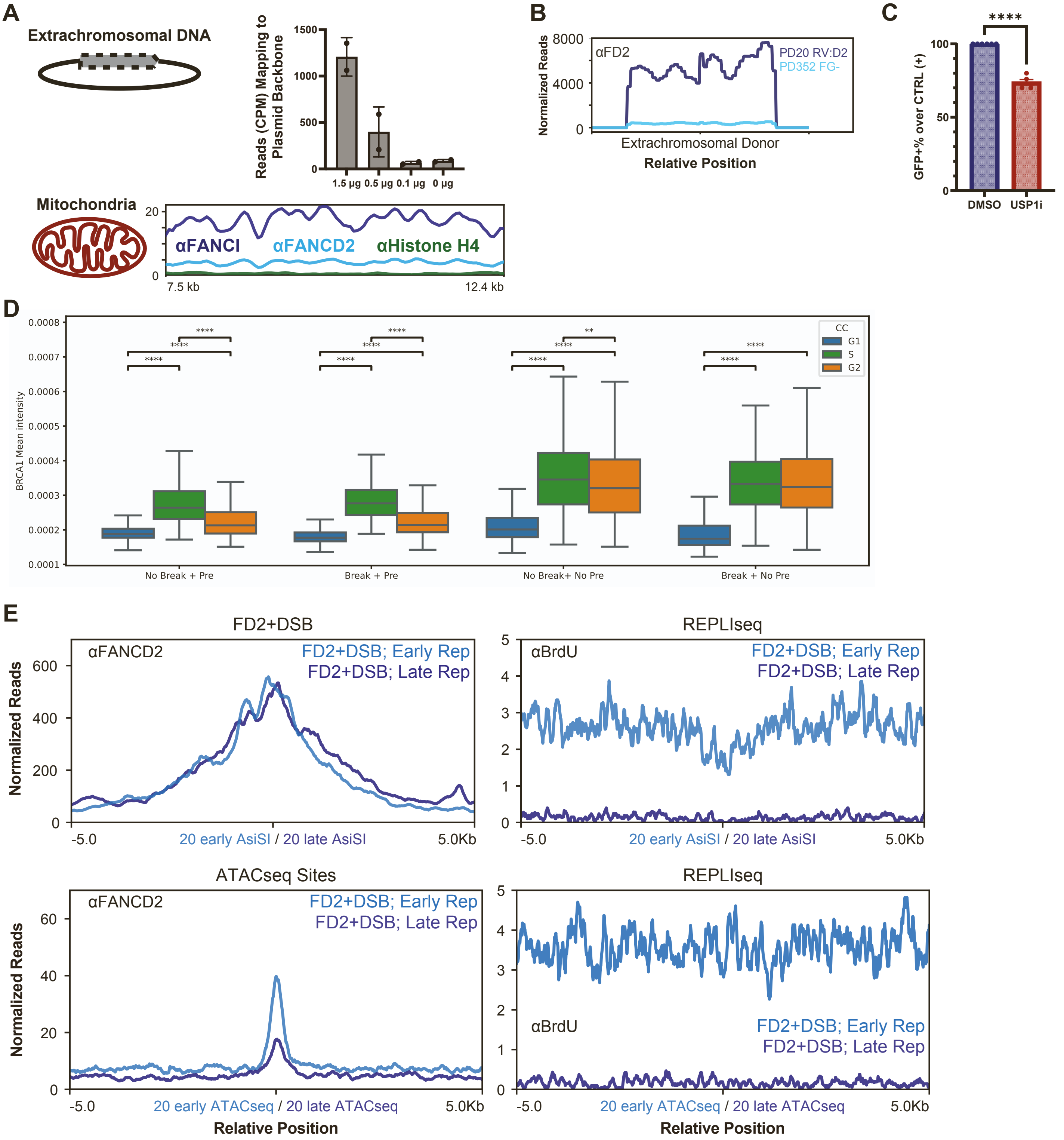

Figure S2:

A. (Top) Total FANCD2 ChIP-seq reads (in counts per million) aligning to the pMB1-ampR plasmid backbone region of extrachromosomal DNA homologous to the HIST1H2BJ or LMNB1 locus in K-562 cells 16 hours after electroporation with Cas9 RNP targeting the HIST1H2BJ locus. (Bottom) ChIP-seq profiles for FANCD2, FANCI, and Histone H4 binding to mitochondrial DNA. Data shown are generated from n=2 biological replicates.

B. ChIP-seq data showing FANCD2 binding to extrachromosomal template DNA in patient-derived fibroblast cell lines expressing FANCD2 (PD20 RV:D2), or lacking the FA core component FANCG (PD352). Data shown are representative of n=2 biological replicates.

C. Normalized recombination (GFP+) percentage at the SceGFP locus in either mock-treated (DMSO) or USP1 inhibitor-treated (USP1i) DR-GFP U2OS cells. Data generated from at least n=3 biological replicates, and the p value was derived from an unpaired t-test, assuming normal (Gaussian) distribution, two-tailed. ****p ≤ 0.0001.

D. QIBC immunofluorescence tracking EdU incorporation, DAPI incorporation, and BRCA1 chromatin binding in pre-extracted (left) or non-extracted (right) DIvA-U2OS cells. Data are presented as BRCA1 mean intensity as a function of cell cycle. Data shown are generated from at least m=12 images and n=2000 cells, and the p values were derived from Shapiro-Wilk test, followed by non-parametric Kruskal-Wallis test, followed by Dunn test. **p ≤ 0.01; ****p ≤ 0.0001.

E. ChIP-seq (left) and REPLIseq (right) data at AsiSI DSB and ATAC-seq open chromatin sites. REPLIseq data is reproduced from PRJNA397123. Data shown are representative of n=2 biological replicates.

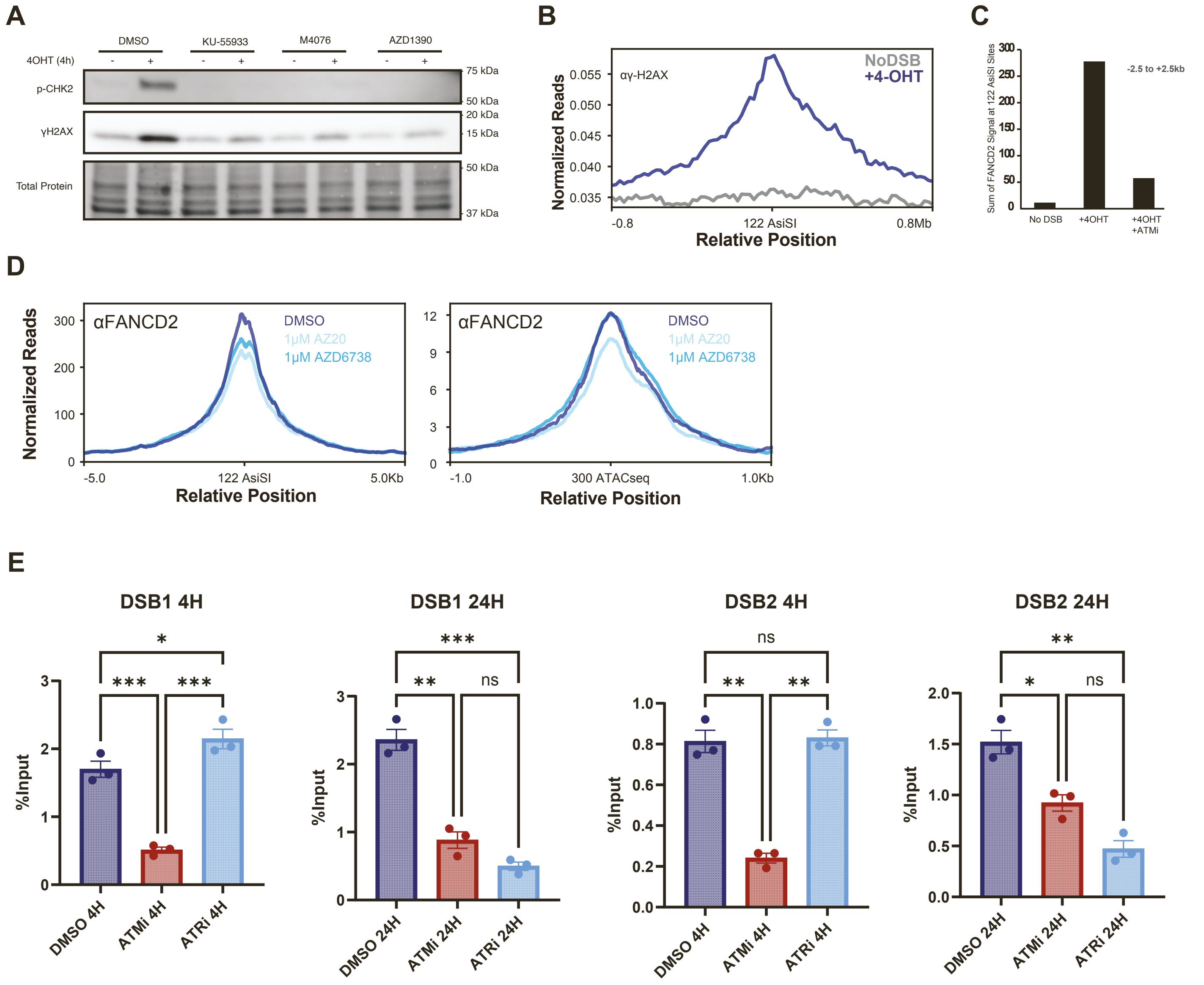

Figure S3:

A. Western blots showing the effect of 4 hours of various ATM inhibitors (10 μM KU-55933, 1 μM M4076, or 0.1 μM AZD1390) on the phosphorylation levels of CHK2 and H2AX in the presence of DNA damage induced by 300 nM 4-OHT. Blots shown are representative of n=3 biological replicates in DIvA-U2OS cells.

B. ChIP-seq data showing γ-H2AX binding to AsiSI sites in U2OS cells after DSB induction. Data shown is reproduced from PRJEB21297 and is representative of n=2 biological replicates.

C. Sum of FANCD2 ChIP-seq signal in a -2.5kb to +2.5kb window spanning 122 AsiSI sites for mock treated (no DSB), DSB-induced (+4OHT), or DSB-induced ATM-inhibited (+4OHT +10 μM KU-55933) conditions. Data shown is the sum of signal from n=2 biological replicates in DIvA-U2OS cells.

D. ChIP-seq summary showing FANCD2 binding to DSBs (left) or open chromatin sites (right) in untreated (DMSO), or ATR inhibitor-treated (1 μM AZ20 or 1 μM AZD6738) conditions. Data shown are representative of n=2 biological replicates in DIvA-U2OS cells.

E. ChIP-qPCR data showing FANCD2 enrichment at two AsiSI DSB sites (DSB1 = chr1:88992917; DSB2 = chr1:109494081) in the presence of ATM (10 μM KU-55933) or ATR (1 μM AZ20) inhibitors 4 hours (4H) or 24 hours (24H) after DSB induction. Immunoprecipitations were performed using FANCD2 antibodies from DIvA-U2OS cells as indicated. Plots are representative of n=3 technical replicates, and the p values were derived from ordinary two-way ANOVA with main effects only, followed by Tukey's multiple comparisons test, with a single pooled variance. *$p \leq 0.05$; **$p \leq 0.01$; ***$p \leq 0.001$.

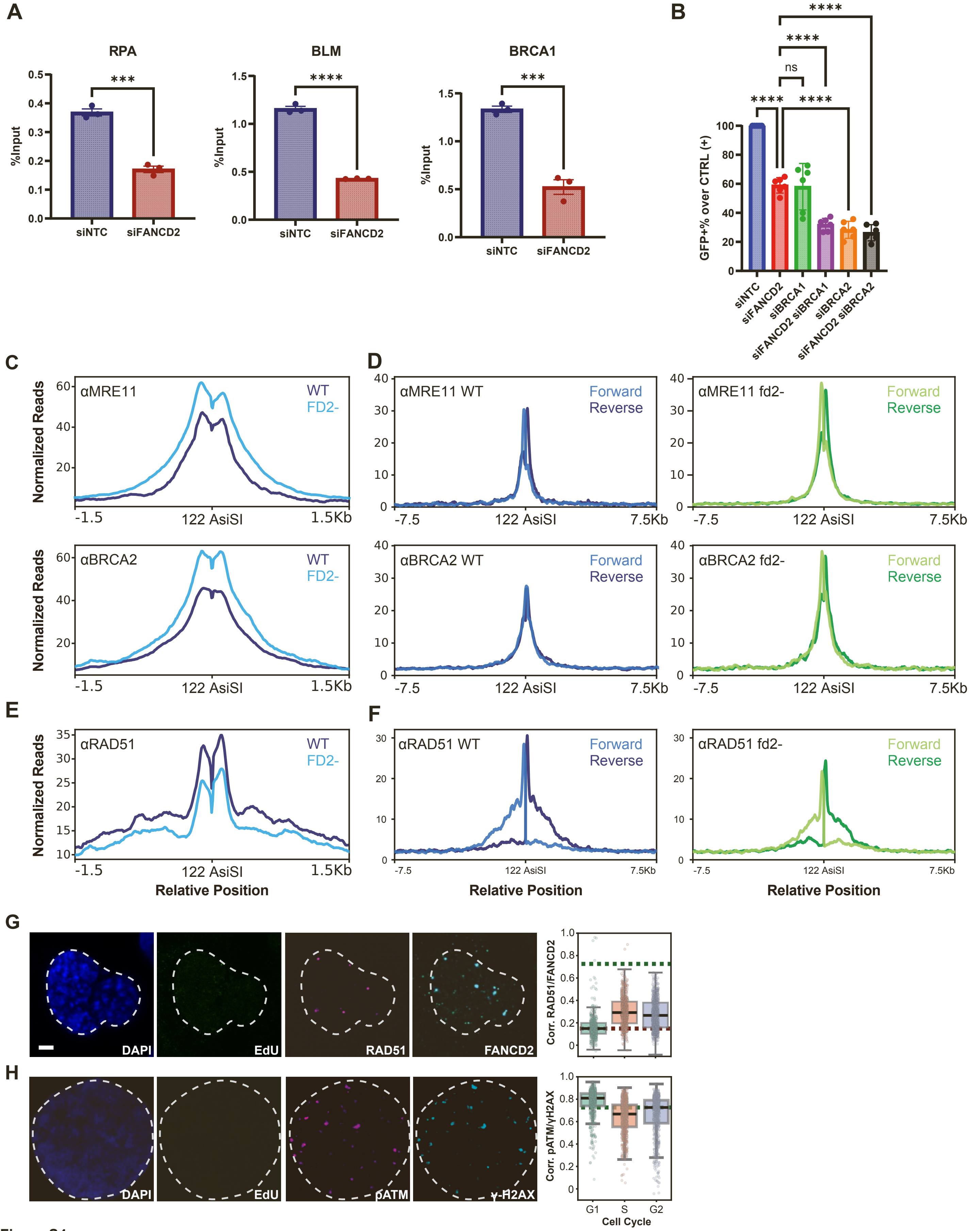

Figure S4:

A. ChIP-qPCR data showing recruitment of RPA, BLM, and BRCA1 to a single AsiSI cut site (chr1:88992917) 4 hours after DSB induction in WT (siNTC) or FANCD2-depleted (siFANCD2) DIvA-U2OS cells. Immunoprecipitations were performed using RPA, BLM, and BRCA1 antibodies from DIvA-U2OS cells as indicated. Plots are representative of n=3 technical replicates, and each p value was derived from an unpaired t-test between siNTC and siFANCD2 conditions, assuming normal (Gaussian) distribution, two-tailed. ***p ≤ 0.001; ****p ≤ 0.0001.

B. Normalized recombination (GFP+) percentage at the SceGFP locus in DR-GFP U2OS cells with the indicated siRNA knockdowns. Data generated from at least n=3 biological replicates, and the p values were derived from ordinary two-way ANOVA with main effects only, followed by Tukey's multiple comparisons test, with a single pooled variance. ****p ≤ 0.0001.

C. ChIP-seq data showing recruitment of MRE11 or BRCA2 to AsiSI DSBs in CRISPRi non-targeting control (WT) and FANCD2 knockdown (fd2-) DIvA-AID-U2OS-ZIM3 cells 4 hours after DSB induction. Data shown are representative of n=2 biological replicates.

D. Stranded ChIP-seq data showing recruitment of MRE11 or BRCA2 to AsiSI DSBs in CRISPRi non-targeting control (WT) and FANCD2 knockdown (fd2-) DIvA-AID-U2OS-ZIM3 cells 4 hours after DSB induction. Data shown are representative of n=2 biological replicates.

E. ChIP-seq data showing recruitment of RAD51 to AsiSI DSBs in CRISPRi non-targeting control (WT) and FANCD2 knockdown (fd2-) DIvA-AID-U2OS-ZIM3 cells 4 hours after DSB induction. Data shown are representative of n=2 biological replicates.

F. Stranded ChIP-seq data showing recruitment of RAD51 to AsiSI DSBs in CRISPRi non-targeting control (WT) and FANCD2 knockdown (fd2-) DIvA-AID-U2OS-ZIM3 cells 4 hours after DSB induction. Data shown are representative of n=2 biological replicates.

G. QIBC data showing colocalization of DAPI, EdU, and RAD51 with FANCD2 in DSB-induced (+300 nM 4-OHT for 24 hours) DIvA-U2OS cells. Example images are presented at left and Pearson correlation is plotted at right. Data shown are generated from at least m=12 images and n=2000 cells. Scale bar = 2 μm.

H. QIBC data showing colocalization of DAPI, EdU, and phospho-ATM with γ-H2AX in DSB-induced (+300 nM 4-OHT for 24 hours) DIvA-U2OS cells. Example images are presented at left and Pearson correlation is plotted at right. Data shown are generated from at least m=12 images and n=2000 cells.

A

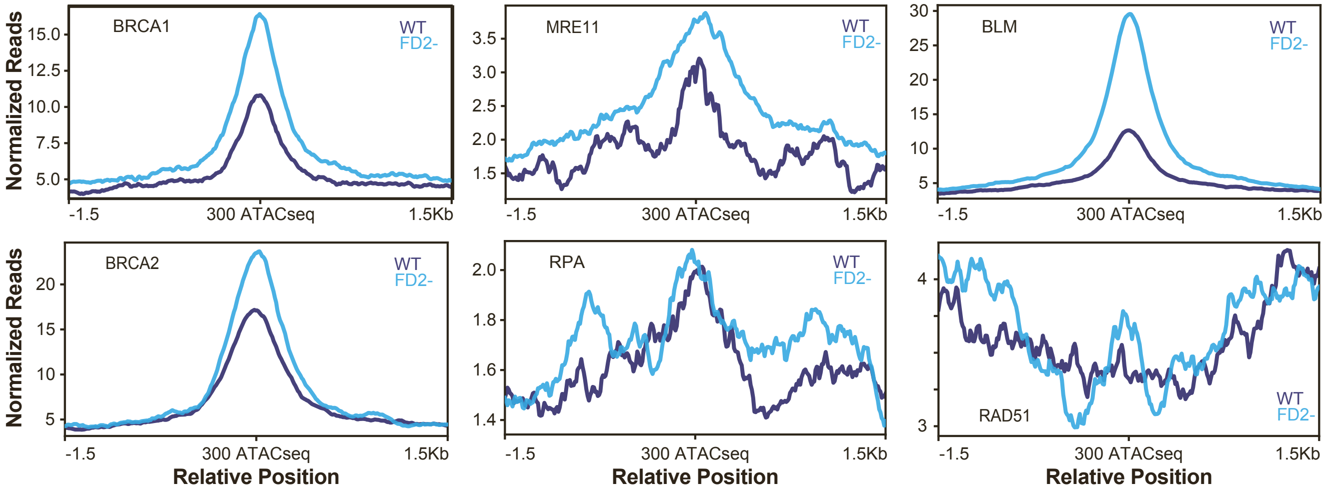

Figure S5:
A. ChIP-seq data showing recruitment of BRCA1, MRE11, BLM, BRCA2, RPA, or RAD51 to open chromatin sites in CRISPRi non-targeting control (WT) and FANCD2 knockdown (fd2-) DIvA-AID-U2OS-ZIM3 cells. Data shown are representative of n=2 biological replicates.

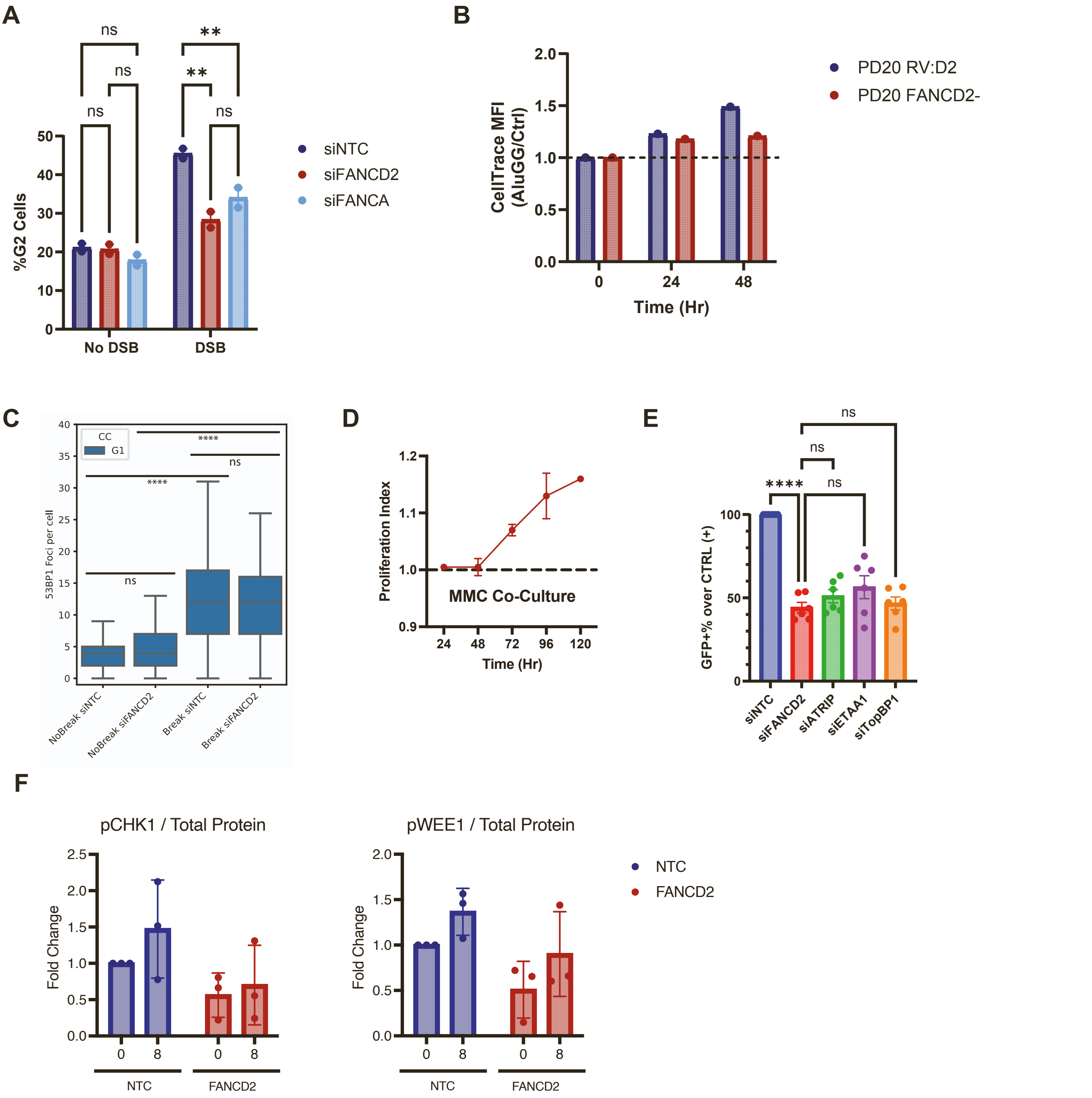

Figure S6:

A. QIBC measurement of G2 abundance in mock (siNTC), FANCD2-depleted (siFANCD2), or FANCA-depleted (siFANCA) DIvA-U2OS cells after 24 hours of mock (noDSB) or 4-OHT (DSB) treatment. Data shown are generated from at least m=12 images and n=2000 cells, and the p values were derived from ordinary two-way ANOVA with full model, followed by Sidak's multiple comparisons test, with a single pooled variance. **p ≤ 0.01.

B. CellTrace signal normalized to control (no DSB) condition at indicated timepoints after DSB induction in patient-derived fibroblast cell lines expressing FANCD2 (PD20 RV:D2), or lacking FANCD2 (PD20). Data shown are generated from n=1 biological replicates.

C. QIBC immunofluorescence tracking EdU incorporation, DAPI incorporation, and 53BP1 foci per G1 cell with and without DSB induction in mock (siNTC) or FANCD2-depleted (siFANCD2) DIvA-U2OS cells. Data shown are generated from at least m=4 images and n=600 cells, and the p values were derived from Shapiro-Wilk test, followed by non-parametric Kruskal-Wallis test, followed by Dunn test. ****p ≤ 0.0001.

D. Co-culture experiments comparing growth rates of CRISPRi non-targeting control (WT) and FANCD2 knockdown (fd2-) DIvA-AID-U2OS-ZIM3 cells continuously grown in 50 ng/mL Mitomycin C (MMC, which induces ICLs). Data shown are generated from n=2 biological replicates.

E. Normalized recombination (GFP+) percentage at the SceGFP locus in DR-GFP U2OS cells with the indicated siRNA knockdowns. Data generated from at least n=3 biological replicates, and the p values were derived from ordinary two-way ANOVA with main effects only, followed by Tukey's multiple comparisons test, with a single pooled variance. ****p ≤ 0.0001.

F. Quantification of Western blots shown in Figure 6E.

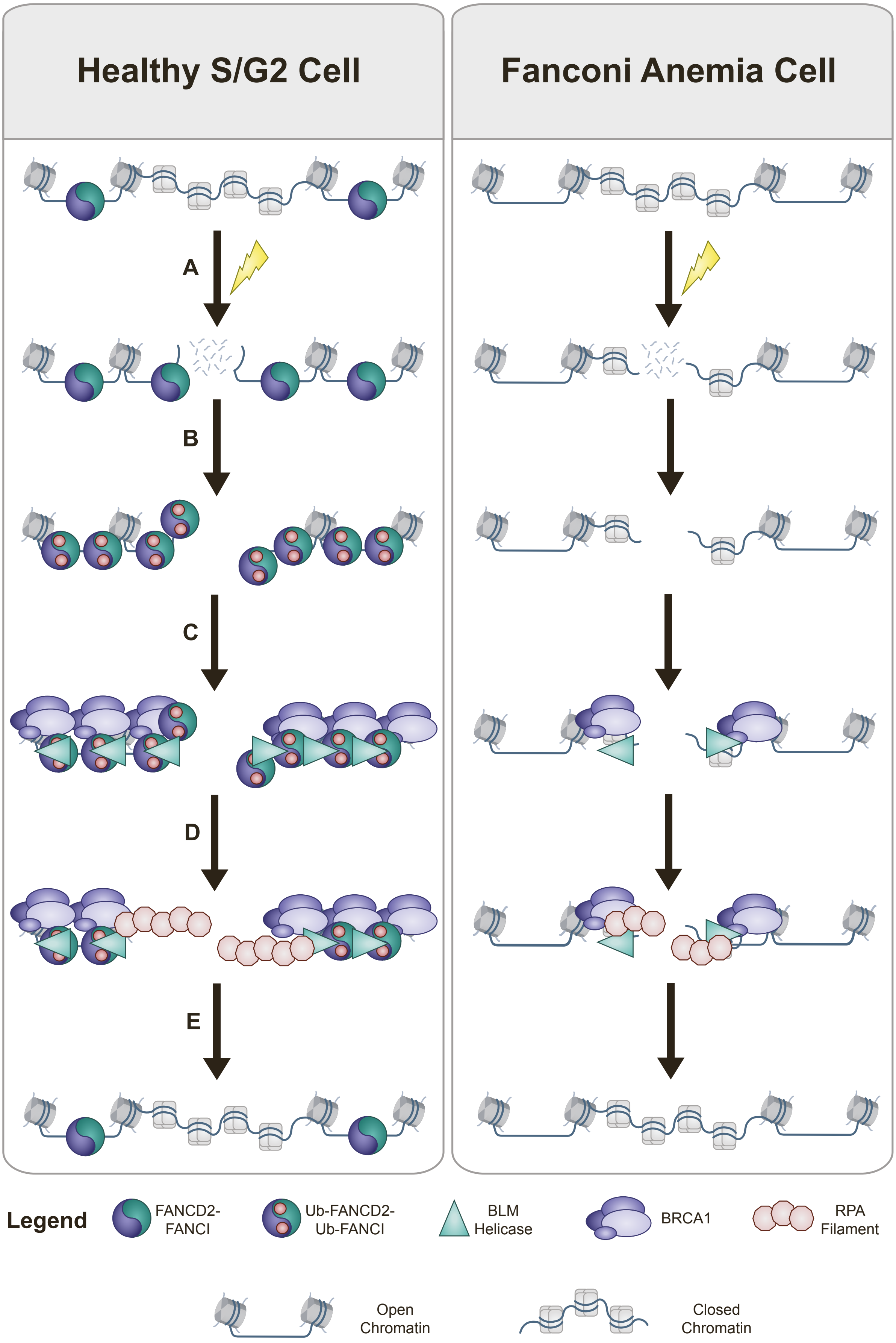

Figure S7:

A. (Healthy S/G2 Cell) The FANCD2-FANCI heterodimer samples sites of open chromatin. DNA damage produces transient DSB-induced open chromatin, leading to the FANCD2-FANCI heterodimer sampling DSBs. (Fanconi Anemia Cell) Cells deficient in FANCD2 cannot form the FANCD2-FANCI heterodimer.

B. (Healthy S/G2 Cell) The FA core complex, an E3 ubiquitin ligase, monoubiquitinates the FANCD2-FANCI heterodimer, decreasing the off rate of the complex. FANCD2-FANCI accumulation at DSBs and nearby open chromatin sites is also dependent on ATM kinase activity and short-range resection activity, facilitated by MRE11. (Fanconi Anemia Cell) Cells deficient in FANCD2 are unable to accumulate FANCD2-FANCI heterodimer at DSBs and nearby open chromatin.

C. (Healthy S/G2 Cell) Resection-induced open chromatin is further stabilized by ubiquitinated FANCD2-FANCI, leading to enhanced loading of the additional resection factors BLM helicase and BRCA1. (Fanconi Anemia Cell) Cells deficient in FANCD2 are unable to stabilize open chromatin during DSB-induced chromatin remodeling, and therefore, load less BLM and BRCA1 to DSBs.

D. (Healthy S/G2 Cell) A G2 arrest provides the cell adequate time for extensive DNA end resection activity, creating single-stranded DNA, which is coated with RPA. (Fanconi Anemia Cell) With less loading of resection factors BLM, BRCA1, and RPA, there is reduced need for an elongated G2 arrest in cells deficient in FANCD2.

E. (Healthy S/G2 Cell) RAD51-mediated homologous recombination repair occurs, resulting in resynthesis of the DNA; post-repair, FANCD2-FANCI is deubiquitinated by USP1 and released from the DNA. (Fanconi Anemia Cell) Cells deficient in FANCD2 repair damage with reduced contributions from BLM/BRCA1/RPA.
